# Forecasting Disease Burden with a Dynamic Transmission Model of Human Papillomavirus and Recurrent Respiratory Papillomatosis in the United States

**DOI:** 10.3390/v16081283

**Published:** 2024-08-11

**Authors:** Cody Palmer, Edith Morais, Joseph Tota

**Affiliations:** 1 Biostatistics and Research Decision Sciences (BARDS), Merck & Co., Inc., Rahway, NJ 07065, USA; 2 MSD, 69007 Lyon, France

**Keywords:** recurrent respiratory papillomatosis, human papillomavirus, vaccination, vertical transmission

## Abstract

Juvenile- and adult-onset recurrent respiratory papillomatosis (JORRP and AORRP) are rare but serious conditions that are caused by oral human papillomavirus (HPV) infections. The proliferation of wart-like growths throughout the respiratory tract can result in medical problems, including death. The current treatment scheme is surgery, though prevention of HPV infection through vaccination is available. A previously developed model for JORRP and AORRP was adapted to the United States using data on disease burden and HPV infection. The model was validated against post-vaccination reductions in disease and used to forecast the future burden of JORRP and AORRP, estimating the impact that HPV vaccination will have on these diseases. Between 2007 (the beginning of HPV vaccination in the US) and 2021, this model estimates that approximately 1393 lives, 22,867 Quality-Adjusted-Life-Years, and over USD 672 million in treatment costs have been saved by HPV vaccination. There is also a substantial reduction in JORRP and AORRP burden, with a 95% reduction in incidence by 2040. Moreover, between 2040 and 2121, the model predicts 3–11 total cases of HPV6/11-related JORRP in the US, and 36–267 total cases of HPV6/11-related AORRP. HPV vaccination in the United States has driven, and will continue to drive, substantial reductions in the public health and economic burden of HPV6/11-related JORRP and AORRP.

## 1. Introduction

Juvenile- and Adult-Onset Recurrent Respiratory Papillomatosis (JORRP and AORRP) are diseases that result from human papillomavirus (HPV) infection and are characterized by the growth of wart-like lesions, which may occur anywhere throughout the respiratory tract, but most often the larynx [1,2]. Patients often face multiple annual surgeries over many years to manage the complications associated with the papillomas, though some will experience a spontaneous remission of the lesions [3,4,5]. AORRP occurs in adults following acquisition of oral HPV infection, while infants and children can progress to JORRP from an HPV infection that is vertically transmitted from an infected mother during labor [6,7,8]. It has been estimated that approximately 1 in 100 prevalent cases of JORRP will die each year [9].

The vast majority (95%) of HPV types detected in papilloma biopsies from JORRP in the United States were either HPV6 or HPV11, types which are targeted by quadrivalent and nonavalent HPV vaccines (4vHPV and 9vHPV, respectively) [3]. As such, significant declines in JORRP incidence have been documented in countries with established HPV vaccination programs that cover HPV6 and HPV11, including the United States and Australia [10,11]. However, national HPV vaccination programs tend to be focused on averting HPV-related cancers. Genital warts, cervical precancers, and JORRP and AORRP are the HPV-related diseases where we will first see the impact of HPV vaccination, which, in part, motivates this modeling study that focuses on JORRP and AORRP. Also, HPV vaccination provides indirect protection for juveniles against vertically transmitted HPV infections, which means that all the benefit that is realized in JORRP reductions is due to this indirect protection, which is another motivating component to this study.

By building on a previous model of HPV [12,13], we aim to quantify two things that, to our knowledge, have yet to be addressed in the literature. The first is to measure the benefit that has already been realized by HPV vaccination in the United States (US) since 2007 in terms of reductions in JORRP and AORRP and gains in quality of life. Other work has been focused on HPV infection, cervical precancers, and genital warts [14,15]. The second is to forecast the burden of HPV6/11-related JORRP and AORRP in the US. Along the way, we will show that our model is able to accurately predict the declines in the birth-cohort incidence of JORRP following HPV vaccination; that most of the currently documented drop in JORRP incidence can be attributed to historical vaccination of girls over the age of 16 since 2007; and that it is very likely that virtually all AORRP and JORRP related to HPV6 and HPV11 (which represents 95% of all JORRP cases) will be eliminated before 2040.

## 2. Materials and Methods

### 2.1. Epidemiological Model Description

A compartmental dynamic transmission model of HPV infection and progression to RRP was adapted to the US, whose fundamental structure has been described elsewhere [16,17]. We will briefly describe the model structure here, and highlight some differences in this model from previous adaptations. The model is structured by age, sex, and sexual activity level. HPV infection can be acquired through sexual mixing between subpopulations at age- and sexual activity- specific rates. Additionally, a fraction of infants are infected at birth at a rate proportional to the weighted prevalence of HPV infection among mothers. This weighting is determined by the proportion of births by age, a feature which was not included in past adaptations.

In the model, as with other dynamic transmission models, sexually acquired HPV infections in a particular age and sexual activity group occur at rates that are proportional to the annual number of sexual partners in that group with members of the opposite sex in another age and sexual activity group and the prevalence of HPV infection in this group. The proportionate factor is generally understood to be the probability of transmission of HPV in a partnership, and is assumed to be uniform across the various age and sexual activity groups. Details on the number of partnerships, as well as other model details, can be found in the Supplementary Material and its sources [18,19,20,21,22,23,24,25,26,27,28,29,30,31,32,33].

HPV infections can either clear or progress to RRP. JORRP and AORRP are differentiated by their age at progression: progression to RRP at 13 years of age or less is considered JORRP (this threshold is chosen to be consistent with data that have been collected on JORRP in the US [3,10]). In the model, we assume that all HPV infections acquired by infants are cleared by the age of 13, so that effectively JORRP is a result of only vertical transmission, whereas all AORRP cases result from sexual transmission of HPV. Populations with RRP will either clear the disease or die, with the possibility that death may be due to non-RRP related causes. Clearance times of HPV infection, JORRP, and AORRP are assumed to follow exponential distributions. However, the clearance rate of oral HPV infections in juveniles is not assumed to be the same as the clearance rate of the sexually acquired infections in adults.

Upon clearance of HPV or RRP, a fraction of the population will seroconvert, providing a degree of protection against subsequent infection. The degree of protection against subsequent infection following clearance is modeled through a reduced chance of infection. These seroconversion rates and degrees of protection are assumed to vary by sex, but not by age. Populations who do not seroconvert have no protection against subsequent infection unless they are vaccinated before exposure.

### 2.2. Differences from Previous Modeling

While this model shares many structural features with the previous adaptations, there are some significant improvements that have been made to better capture the features of HPV, JORRP, and AORRP. Progression from HPV infection to JORRP is assumed to vary by age, meaning that longer-duration infection will be more likely to progress. This is in contrast to the age-independent progression rates of previous models. In this model, we also calibrated the clearance rate of JORRP and AORRP against cohort data (see Section 2.4), as opposed to an assumption of 5 years. We will also be estimating JORRP- and AORRP-specific outcomes (e.g., surgeries and annual costs) on an annual basis, rather than assuming a lifetime total.

### 2.3. Vaccine Model Description

**Figure 1:**
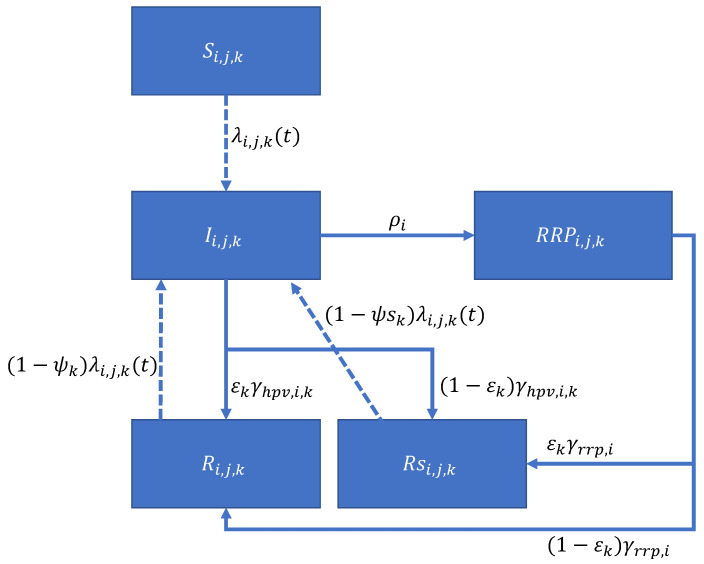
A simplified flow diagram of the compartmental model. *i* represents the age index, *j* the index for sexual activity, and *k* is the sex. *S* is susceptible, *I* HPV-infected, *R* is recovered without seroconversion, Rs is recovered with seroconversion. $\lambda_{i,j,k}\left(t\right)$ is the force of infection, $\rho_{i}$ is the age-specific progression rate to RRP, $\psi_{k}$ and $\psi s_{k}$ are the sex-specific degrees of protection against subsequent infection, $\gamma_{hpv,i,k}$ and $\gamma_{rrp,i}$ are the age-specific clearance rates of HPV and RRP, respectively. $\varepsilon_{k}$ is the fraction of cleared infection/disease that seroconverts.

HPV vaccination using 4vHPV or 9vHPV vaccine is also simulated in the model, and the impact of HPV vaccination is modeled in two ways: prevention of sexually transmitted HPV infection, and reduced progression to AORRP in breakthrough infections acquired after vaccination. As a conservative assumption, we do not assume that children of vaccinated mothers have any intrinsic protection against infection. Indirect protection for infants comes from vaccination and subsequent protection against infection of the mother, but infants have no protection from a breakthrough infection in a vaccinated mother. While there is some evidence to suggest that there is placental transfer of antibodies following vaccination [34], there is no indication whether the effect is protective or is present in women vaccinated prior to pregnancy. However, if children of vaccinated mothers do have some protection, then our results will underestimate the benefit of HPV vaccination on JORRP. A simplified flow diagram of the model is given in Figure 1. Further details can be found in the Supplementary Materials.

### 2.4. Model Calibration

#### 2.4.1. Calibrated Parameters

The data used to parameterize the model come from various sources. Demographic data and sexual behavior data are the same as were used in a previous adaptation of this model [12] and are given in the Supplementary Materials. Several parameters did not have specific estimates that could be used to directly parameterize the model, and so some relevant target data were collected for calibration of those parameters. The calibrated parameters are listed here:

- Probability of vertical transmission of an HPV infection.
- Probability of transmission of an HPV infection in a sexual partnership.
- Age-specific rates of HPV infection clearance.
- Sex-specific proportion of cleared HPV infections that will seroconvert and provide protection against subsequent infection.
- Sex-specific degree of protection given by a cleared, seroconverted HPV infection.
- Age-specific rates of progression to JORRP and AORRP.

Further details on these parameters, along with prior ranges is available in the Supplementary Materials. All data were collected from before HPV vaccination was available, and we assumed that they represented an estimate of the steady, endemic state of HPV infection, JORRP, and AORRP in the United States.

#### 2.4.2. Target Data for Calibration

To calibrate HPV transmission-related parameters, we used age-specific HPV6/11 female prevalence and male seroprevalence data from NHANES [35,36].

It is worth noting here that the model allows, through vertical transmission, the infection of infants, and the data used in these HPV infection targets were for adults. The infant/child HPV infection-related and RRP-related parameters were calibrated against JORRP birth cohort incidence, age of diagnosis, and HPV-type distribution data [3,10]. Adult RRP progression rates were calibrated against estimates of AORRP incidence in the US [1]. Finally, clearance parameters for JORRP and AORRP were estimated from data on a cohort of Norwegian patients with RRP [37]. The point estimates were produced by assuming exponential wait times for RRP clearance and finding the maximum likelihood estimator for the median and interquartile range of the cohort data. Further details can be found in the Supplementary Materials.

#### 2.4.3. Calibration Methods and Posterior Analysis

With these data, Bayesian inference was used to produce 500 samples from posterior distributions of the various parameters. The posterior distributions of the calibrated parameters were analyzed for the purposes of model criticism, and issues surrounding the identifiability of the parameters were investigated. In particular, we used Pearson’s correlation coefficient $\hat{\rho }$ to identify linear relationships between the calibrated parameters. This was performed to quantify the degree to which the calibrated parameters are identifiable from the data used for calibration. The target data, methodology, prior ranges, posterior distributions, fits, and diagnostics for the Markov Chain Monte Carlo algorithm are given in the Supplementary Materials. The scenarios below are run for each of these 500 posterior parameter samples.

Because these 500 parameter sets are drawn empirically, the ranges cannot be used to give an appropriate confidence interval, according to a mathematically rigorous understanding of the term. As such, we will designate the central 95% of the posterior ranges as the 95% credibility interval; meaning that 95% of the parameters that produce credible outputs relative to the calibration targets will be within this interval. This use of the term is consistent with other infectious disease modeling (see, for example, [38]).

### 2.5. Model Outcomes

After calibration, historical data on 4vHPV and 9vHPV vaccination data were integrated to predict the impact of vaccination on JORRP and AORRP in the US since the beginning of the vaccination program as well as into the future. The 4vHPV or 9vHPV vaccine is assumed to provide direct protection against AORRP in two ways. First, it provides a degree of protection against HPV6 and HPV11 infection. In the event of a breakthrough infection, we assume that vaccination using 4vHPV or 9vHPV vaccine will provide a degree of protection against progression of the infection to AORRP due to HPV 6 and HPV11. The degree of protection against HPV6/11 infection is based on the results from clinical trials, and the protection against progression to RRP is assumed to be equal to that of genital warts (since no trial has been sufficiently powered to identify protection against AORRP). This assumption is consistent with previous modeling, but as a sensitivity test, we ran scenarios where no protection against progression of infection to AORRP is assumed. Due to direct protection against HPV infection in adults, infants will experience indirect protection against vertical transmission of HPV 6/11.

We collected various predicted outcomes for these scenarios including:

- Estimated incidence of JORRP in two-year birth cohorts from 2006–2007 to 2012–2013.
- Population incidence of AORRP and JORRP over the full time horizon.
- Amount and discounted costs of AORRP- and JORRP-related surgeries.
- Discounted Quality Adjusted Life Years (QALYs) lost due to RRP morbidity and mortality.

The birth-cohort incidence for JORRP up to 2013 was collected for the purpose of validating the model through comparison of these outcomes with the measured drops in birth-cohort incidence in other published work [10]. The population incidence, in addition to measuring vaccine effectiveness over time, is also used to estimate the number of annual cases in the US. The health-economic outcomes are collected to measure the medical and economic burden that has been relieved by HPV vaccination from 2006 to now. The costs and QALYs are estimated in the model by computing the total time spent by the population in the RRP state. To compute the number of surgeries, this number is multiplied by the annual number of surgeries, while for QALYs and costs, the amount of time is discounted by 3% in the integrand and then multiplied by the appropriate disutility or surgical costs. Values and formulas for these computations can be found in the Supplementary Materials.

### 2.6. Model Scenarios

We considered three vaccination scenarios to help understand the past and future impact of HPV vaccination in the United States:

- Status Quo—Vaccination using 4vHPV or 9vHPV vaccine continues at the most recent rates
- High Vaccination Coverage Rate (VCR)—Vaccination using 4vHPV or 9vHPV vaccine with 90% coverage.
- Vaccination Age Counterfactual Scenario—vaccination using 4vHPV or 9vHPV vaccine of individuals over 16 years old is removed from the historical and future vaccination rates.

The first two scenarios are straightforward. The third scenario is intended to measure how much of the drop in JORRP incidence that has been seen at the onset of the HPV vaccination program in the US was due to the inclusion of these age groups in the program, as opposed to a pre-sexual-debut adolescent population only.

## 3. Results

### 3.1. Calibration Results, Inferred Parameters, and Future Directions for Clinical Data Collection

Full posteriors for the parameter values are available in the Supplementary Materials, but the mechanism of impact for HPV vaccination on JORRP is highlighted through the inferred probability of vertical transmission of HPV infection. A mean estimate of 84.44% (95% credibility interval (CI): (59.99%, 100%)) of infected mothers will transmit their HPV6 infection vertically. HPV11 infections appear to be less likely to be transmitted, with a mean of 38.55% (CI: (20.40%, 61.69%)) of maternal infections being transmitted.

Progression to JORRP was allowed to vary by age in the calibration to achieve the targeted distribution of age-of-diagnosis. The calibrated rates differed by orders of magnitude among different age groups (see Supplementary Materials). Since the age structure in the model is explicit, and there is no possibility of HPV infection in age groups under the age of 13 through sexual contact, the increasing rates calibrated here suggest that the hazard function for RRP progression is not constant over the infectious period. The implications that this has for modeling JORRP and AORRP is deferred to the Section 4.

Correlations between parameters in the posterior samples were explored to help elucidate how future clinical data estimating specific parameters (which would narrow prior ranges) may improve posterior estimates. Several pairs of parameters exhibited a nearly linear relationship ($|\hat{\rho }|\;\geq \;0.7$) across the samples from the posterior, some of which we highlight here, while the rest are presented in the Supplementary Materials. The probability of vertical transmission showed the strongest correlation with a progression rate of HPV6 infection to RRP from age 0 to 3 ($\hat{\rho }=-0.89$) and a clearance rate of HPV among juveniles ($\hat{\rho }=0.73$). These correlations were also seen in HPV11 ($\hat{\rho }=0.81$ and $\hat{\rho }=0.79$, respectively). The strongest correlations observed in HPV6 were among the degree of protection against infection imparted by cleared natural infections with seroconversion and the proportion of cleared infections that seroconvert, among both males and females ($\hat{\rho }=-0.93$ and $\hat{\rho }=-0.89$, respectively), suggesting that precise clinical estimates of seroconversion proportions will significantly narrow the posterior estimates of degree of protection imparted by natural infection derived from this model. Interestingly, these degree of protection/seroconversion correlations were not seen in the posterior samples of HPV11 parameters.

### 3.2. Model Validation and Counterfactual Analysis: 2004–2013 Birth Cohort Incidence

**Figure 2:**
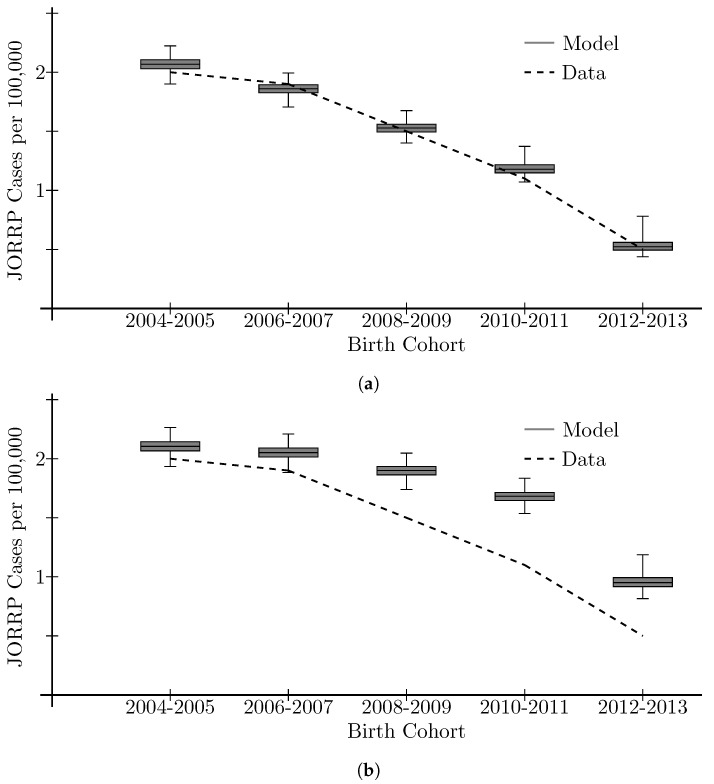
Model validation against real world data, using different assumptions of VCR. (**a**) NIS–TEEN and NHANES VCR data. (**b**) Removal of vaccination for populations ≥16 years of age.

As previously mentioned, the model used birth cohort incidence of JORRP in 2004–2005 (prior to vaccination) as a calibration target. However, in the study where these data were collected [10], the incidence of JORRP in four subsequent two-year birth cohorts (2006–2007, 2008–2009, 2010–2011, 2012–2013) was also collected, and showed a substantial drop from 2 per 100,000 in the 2004–2005 birth cohort to 0.5 per 100,000 in the 2012–2013 birth cohort (see Figure 2a).

As a validation of the model, we predicted, using vaccination coverage data from NIS-TEEN and NHANES (see Supplementary Materials), the birth-cohort incidence over this time period to compare with the aforementioned data. We plot the range of model outputs in Figure 2a as boxplots. Visually, the model accurately captures the downward trend in birth-cohort JORRP incidence in the US. To quantify the error between the model predictions and the data, we computed a mean relative error, across all the calibrated parameter sets, of 0.062.

A counterfactual scenario was considered to measure the impact of vaccination of individuals over the age of 16 years on these observed drops in JORRP incidence. A visual comparison is given in Figure 2b. The impact of vaccination of the population over the age of 16 years has its largest impact in the 2008–2009 and 2010–2011 birth cohorts, where an estimated 80.1% and 64.7% of the drop in JORRP incidence can be attributed to this population receiving HPV vaccination (see Supplementary Materials). In the 2012–2013 birth cohort, this proportion goes down to an estimated 30.1% as younger, vaccinated females move into child-bearing age.

### 3.3. Economic Results: 2007–2021

From 2007 (the onset of HPV vaccination) to 2021, the model estimates that there has been a 55% (CI: (51%, 58%)) reduction in the number of RRP-related surgeries among juveniles, and thus in surgical costs. The reduction in surgeries and surgical costs among adults is even more significant at 62% (CI: (56%, 66%)). Total numbers of averted surgeries and costs (compared to no vaccination) are presented in Table 1. To compare these economic results against other modeling work, we used the model to estimate the lifetime cost and lifetime QALYs lost per case of JORRP or AORRP, discounted at 3% from onset of JORRP or AORRP. These outcomes were then compared to inputs used in earlier modeling work on the economic benefit of HPV vaccination of JORRP [39]. The current model estimated average lifetime QALYs lost per JORRP or AORRP case at 1.8 QALYs, compared to the 1.05 (0.33–3.05) QALY loss used in [39]. While the QALYs lost were comparable between the two models, there was a significant difference in the estimates of the lifetime cost of a JORRP or AORRP case at USD 51,997 in our model to USD 198,500 (105,000, 357,000) in [39], when the same cost per surgery was used in both models. This difference is explained by the number of assumed surgeries per year: 2–3 in this modeling work [3], compared to 7 in [39]. If the annual number of surgeries were scaled up to 7 in our model, lifetime cost per JORRP or AORRP case would be comparable.

As a sensitivity analysis, we also considered the averted surgery costs under different estimates. Bishai et al. [9] used a range from USD 3500 to 5249 (USD 1996) per surgery for sensitivity analyses. Under these estimates the total surgical costs avoided since 2007 varied from USD 488.6 M to USD 736.1 M.

A probabilistic sensitivity analysis was run on the QALYs saved when the utility associated with JORRP and AORRP is varied and 95% of the draws were between 7777.65 and 49,769.2 QALYs saved between 2007 and 2021. Further details can be found in the Supplementary Materials.

### 3.4. Forecasted JORRP and AORRP Incidence and Mortality

**Figure 3:**
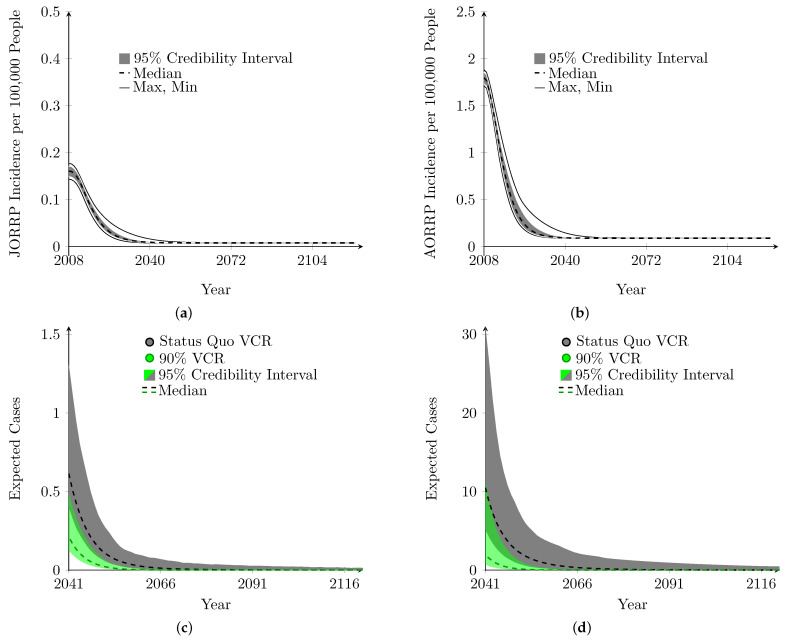
Forecasted RRP incidence and total cases attributable to HPV6/11 in the US to 2124. (**a**) JORRP. (**b**) AORRP. (**c**) JORRP cases after 2040. (**d**) AORRP cases after 2040.

The model was used to forecast birth cohort incidence of JORRP and AORRP. The predicted incidence for JORRP and AORRP is given in Figure 3. The model results indicate that after 2040, RRP will be reduced by nearly 95%, and almost all cases will be due to HPV types not covered by the vaccine. To emphasize this, we have produced the predicted number of new cases of RRP in both adults and juveniles by year after 2040. For JORRP, we can see that the number of cases falls below one by 2043, suggesting a non-trivial probability that no cases may be seen that year and multiple years may pass before new cases emerge. Accumulating the risk from 2040 through 2121, we can expect a total of 5 (3, 11) new cases of JORRP in the United States that could be attributed to the vaccine types. Similar computations for AORPP suggest that from 2040 onward, we may only see 86 (36, 267) cases of AORRP that can be attributed to the vaccine types. The reason that this number is larger than what is seen in JORRP is not due to a substantial higher risk of developing AORRP but is rather due to the larger size of the adult (13+) population. JORRP and AORRP mortality averted is significant across the time horizon. With a total of 28,255 (27,297, 29,212) lives saved from 2007 to 2121, 1393 of these lives saved occurred before 2022. The model predicted 0.766 (0.532, 1.705) JORRP deaths due to HPV6/11 after 2040, suggesting it is highly unlikely that we will see more than 2 deaths due to HPV6/11-related JORRP after 2040. AORRP deaths due to HPV6/11 after 2040 were estimated to be 47.5 (37.4, 81.5).

To understand the impact of the assumed efficacy against RRP progression for breakthrough infection among vaccinated individuals, we set this efficacy to 0 and ran the model. The results for JORRP remained unchanged since we do not assume an increased probability of transmission among the subpopulation with RRP. Among adults, because of the rare chance of RRP progression when infected, the number of cases averted by efficacy against RRP progression was small compared to the total number of cases (see Supplementary Materials).

### 3.5. Higher VCR and Expected RRP Cases over Time

In terms of incidence of RRP at the population level, vaccination at a higher rate had very little effect. However, when we look at the absolute number of cases after 2040, we can see that higher vaccination rates have a significant impact; we expect a total of 25 (9, 136) AORRP cases after 2040, and a total of 3 (2, 6) JORRP cases after 2040 (see Figure 3c,d). By increasing vaccination coverage to 90% there is an acceleration of the decline in RRP cases that results in a shortening of the timeline: the results achieved in 2040 by maintaining current vaccination levels are achieved by 2033 with 90% VCR.

## 4. Discussion

HPV vaccination has been identified as a cost-effective strategy for the control and prevention of HPV-related anogenital and oropharyngeal cancers (e.g., [40]). Indeed, in some contexts, HPV vaccination may be cost-effective on the basis of averting these cancers alone, apart from any other HPV-related disease like RRP or genital warts [41,42]. Thus, our modeling study has looked beyond cost-effectiveness and into other public health and health economic considerations.

The first concern that arises in a modeling study is whether the modeling approach is valid. We have used an established compartmental model for this assessment that is calibrated to, and otherwise parameterized with, US-specific data. Additionally, we used the model to predict the decrease in birth-cohort incidence of JORRP in the US for 2004 to 2013, and found that our prediction closely matched reality, suggesting that the model can accurately capture the dynamics and natural history of HPV infection and RRP disease. Moreover, we used this to show that a significant proportion of the benefit, in terms of JORRP reduction, that is realized so soon after the onset of the HPV vaccination program comes from the fact that girls over the age of 16 have been vaccinated.

Additionally, the calibration of the model provided insights into the natural history of HPV6 and HPV11 infection, the mechanism of vertical transmission of HPV 6 and HPV 11 infections, and the natural history of JORRP. The probability of vertical transmission of HPV6 was found to be higher (≥60%) than pooled estimates across various HPV types (see [43] and its sources, in particular [44,45]). However, credibility intervals for HPV11 were more consistent with these pooled estimates (38.55% in our model vs. 27.4% in [44], but still much higher than the 8.9% of [43]). However it is not immediately clear from the cited data that our model estimates contradict them, since they are not split by specific HPV types. It could be that HPV6 and HPV11 are not sufficiently represented in these pooled sets for us to expect consistent estimates between these data and the model.

The model calibration showed that the rate of progression to JORRP varied by age, suggesting that in order to reproduce the data on the age of diagnosis (assuming diagnosis corresponds to progression), the hazard of progression to JORRP needs to increase over time. This has implications for modeling, since it suggests that the standard approach of assuming exponentially distributed wait-times for infection to JORRP is not correct. As such, we cannot necessarily rule out the possibility that the same could be true for AORRP. Hence, the structure of any future model should take into account the time since infection in order to capture the non-constant hazard function [46].

While the uncertainty around the various model parameters may be significant, one thing that is certain is that HPV vaccination has been, currently is, and will continue aggressively driving down the incidence of HPV6/11-related JORRP and AORRP in the US. We will now summarize and discuss the model results for both the past and future.

Looking back, between the years of 2007 and 2021, our modeling analysis suggests that the HPV vaccination program in the US has averted, compared to no vaccination, a substantial economic and public health burden. Nearly 1400 lives have been saved, and over 168,000 surgeries averted in this time period, resulting in savings of over half a billion US dollars. These may even be significant underestimates, as was previously discussed (Section 3.3), since the literature presents somewhat larger estimates of the number of surgeries per year than was used in this analysis. Also, these cost estimates may be conservative since we did not account for the possibility of malignant transformation of RRP (see [47], for example). Because of the grave nature of RRP, the amount of QALYs gained since 2007 is over 22 thousand, which leads to a per-case QALY loss estimate that is consistent with the literature (see Section 3.3).

Looking into the future of AORRP and JORRP in the US, for all the parameter sets that we inferred, and all the scenarios we considered, the incidence of JORRP and AORRP decreases by nearly 95% by the year 2040 (i.e., almost all of the RRP attributable to HPV6 and HPV11). Thus, this reduction is achieved regardless of the uncertainty in:

- The natural history of HPV infection.
- The probability of vertical transmission.
- The natural history of AORRP and JORRP.
- The efficacy of the vaccine against progression to RRP.

The forecasted population level incidence did not appear to change much when VCR was increased to 90%. However, the effect of this increase in VCR is best demonstrated in the absolute number of expected cases over time. In this sense, the tails of the curves are where the change to 90% VCR is most clearly seen. This is made apparent in Figure 3c,d, and by the 7-year acceleration in the reduction in cumulative cases of HPV6/11-attributable AORRP and JORRP. Furthermore, previous modeling studies have suggested that high VCR may be a key part of eradicating HPV6 and HPV11, thus permanently eliminating the causative agent for 95% of AORRP and JORRP [48].

Much of what we have discussed to this point has highlighted the strengths of our analysis: Validation of the model against historical data; agreement of results and estimates with the literature; the consistency of forecasted results in the face of uncertainty. However, as with any modeling study, there are some limitations to our results.

These limitations fall into two broad categories: limitations due to the model structure, and limitations due to the time horizon. In the first category would fall our various structural assumptions and model parameter values. HPV transmission is mediated by sexual behavior and practices that are not explicitly modeled here, which could make our estimates of the transmission probability (both vertical and sexual) more uncertain. Moreover, we have assumed closed sexual networks, that exclude the importation of infected, sexually active people.

We assume a relatively simple disease course for RRP (AORRP in particular), and the exponential wait-times for progression and clearance may allow for a larger variance in wait-times than what may actually be the case, meaning that the model may be not accurately estimating the amount of time spent in the RRP state. Further data and research (perhaps with mechanistic models [49,50]) are needed to assess the quality of this assumption.

The second class of limitations is associated with the long time horizon of the study, and the fact that we have assumed that many facets of HPV epidemiology and control will remain constant over this time period. Sexual behavior, in terms of age, number of partners, preferences, and practice, may change substantially over short periods of time; increases in sexual behavior may increase the amount of time to reduce JORRP and AORRP, while decreases in sexual activity may accelerate it. We have also assumed that vaccination coverage and vaccine effectiveness remain constant as we project into the future. However, decreases in vaccine coverage may allow for a resurgence in HPV6/11-related JORRP and AORRP.

It is difficult to know how changes in the prevalence of HPV types that are not covered by the 4vHPV or 9vHPV types over the time horizon may affect our results. Less evidence exists regarding JORRP and AORRP cases that may be caused by these other types [3]. However, some studies suggest there is no evidence of type replacement [15], meaning that increases in JORRP and AORRP due to these other types may be unlikely.

Finally, since we did not project averted surgeries, costs, or QALYs gained into the future, we have no results that would be impacted by changes to treatment pathways or costs (for example, the use of HPV vaccination as an adjuvant therapy [51]).

## 5. Conclusions

HPV vaccination is rapidly decreasing incidence of RRP among both adults and juveniles, and our modeling suggests that RRP due to HPV6/11 may vanish within the next two decades. Continued vaccination will also produce a tangible economic benefit, reducing annual costs of RRP treatment while producing considerable quality of life gains.

## Figures and Tables

**Table 1:** Averted surgeries and surgical costs, and QALYs saved from 2007 to 2021.

	Averted Surgeries	Averted Surgical Costs (USD, 2007) ^1^	QALYs Saved ^2^
JORRP (≤13)	5800	22.5 M	–
AORRP (>13)	162,338	650 M	–
RRP	168,138	672.5 M	22,847.5

^1^ Approximately 18% of the USA population is aged 13 or less, explaining the significant difference in the magnitude of averted surgeries and costs. ^2^ Prevalent RRP cases are not differentiated in the model by source of infection; therefore, QALYs saved by age is aggregated for both JORRP and AORRP.

## Data Availability

Original contributions presented in the study are included in the article/Supplementary Material, further inquiries can be directed to the corresponding author.

## Supplementary Materials

The following supporting information can be downloaded at: https://www.mdpi.com/article/10.3390/v16081283/s1.

## Abbreviations

The following abbreviations are used in this manuscript:

RRP	Recurrent Respiratory Papillomatosis
JORRP	Juvenile-Onset Recurrent Respiratory Papillomatosis
AORRP	Adult-Onset Recurrent Respiratory Papillomatosis
HPV	Human Papillomavirus
4vHPV	Quadrivalent HPV Vaccine
9vHPV	Nonavalent HPV Vaccine
VCR	Vaccination Coverage Rate
QALY	Quality-Adjusted Life Year
NHANES	National Health and Nutrition Examination Survey

## References

[1] Derkay C.S. (1995). Task force on recurrent respiratory papillomas: A preliminary report. Arch. Otolaryngol.-Head Neck Surgery.

[2] Derkay C.S., Wiatrak B. (2008). Recurrent respiratory papillomatosis: A review. Laryngoscope.

[3] Amiling R., Meites E., Querec T.D., Stone L., Singh V., Unger E.R., Derkay C.S., Markowitz L.E. (2021). Juvenile-onset recurrent respiratory papillomatosis in the United States, epidemiology and HPV types—2015–2020. J. Pediatr. Infect. Dis. Soc..

[4] Ivancic R., Iqbal H., deSilva B., Pan Q., Matrka L. (2018). Current and future management of recurrent respiratory papillomatosis. Laryngoscope Investig. Otolaryngol..

[5] Derkay C.S., Bluher A.E. (2019). Update on recurrent respiratory papillomatosis. Otolaryngol. Clin. N. Am..

[6] Koskimaa H.M., Waterboer T., Pawlita M., Grénman S., Syrjänen K., Syrjänen S. (2012). Human papillomavirus genotypes present in the oral mucosa of newborns and their concordance with maternal cervical human papillomavirus genotypes. J. Pediatr..

[7] Lacey C.J., Lowndes C.M., Shah K.V. (2006). Burden and management of non-cancerous HPV-related conditions: HPV-6/11 disease. Vaccine.

[8] Syrjänen S., Rintala M., Sarkola M., Willberg J., Rautava J., Koskimaa H., Paaso A., Syrjänen K., Grénman S., Louvanto K. (2021). Oral Human Papillomavirus Infection in Children during the First 6 Years of Life, Finland. Emerg. Infect. Dis..

[9] Bishai D., Kashima H., Shah K. (2000). The Cost of Juvenile-Onset Recurrent Respiratory Papillomatosis. Arch. Otolaryngol.-Head Neck Surg..

[10] Meites E., Stone L., Amiling R., Singh V., Unger E.R., Derkay C., Markowitz L.E. (2021). Significant Declines in Juvenile Onset Recurrent Respiratory Papillomatosis following HPV Vaccine Introduction in the United States. Clin. Infect. Dis. Off. Publ. Infect. Dis. Soc. Am..

[11] Novakovic D., Cheng A.T., Zurynski Y., Booy R., Walker P.J., Berkowitz R., Harrison H., Black R., Perry C., Vijayasekaran S. (2018). A prospective study of the incidence of juvenile-onset recurrent respiratory papillomatosis after implementation of a national HPV vaccination program. J. Infect. Dis..

[12] Daniels V., Prabhu V.S., Palmer C., Samant S., Kothari S., Roberts C., Elbasha E. (2021). Public health impact and cost-effectiveness of catch-up 9-valent HPV vaccination of individuals through age 45 years in the United States. Hum. Vaccines Immunother..

[13] Daniels V., Saxena K., Roberts C., Kothari S., Corman S., Yao L., Niccolai L. (2021). Impact of reduced human papillomavirus vaccination coverage rates due to COVID-19 in the United States: A model based analysis. Vaccine.

[14] Drolet M., Bénard É., Pérez N., Brisson M., Ali H., Boily M.C., Baldo V., Brassard P., Brotherton J.M., Callander D. (2019). Population-level impact and herd effects following the introduction of human papillomavirus vaccination programmes: Updated systematic review and meta-analysis. Lancet.

[15] Checchi M., Mesher D., Panwar K., Anderson A., Beddows S., Soldan K. (2023). The impact of over ten years of HPV vaccination in England: Surveillance of type-specific HPV in young sexually active females. Vaccine.

[16] Elbasha E.H., Dasbach E.J. (2010). Impact of vaccinating boys and men against HPV in the United States. Vaccine.

[17] Elbasha E.H., Dasbach E.J., Insinga R.P. (2007). Model for assessing human papillomavirus vaccination strategies. Emerg. Infect. Dis..

[18] (2022). Teen Vaccination Coverage Publications and Resources. https://www.cdc.gov/vaccines/imz-managers/coverage/teenvaxview/pubs-presentations.html.

[19] Abma J.C. (2001). Sexual Activity and Contraceptive Practices among Teenagers in the United States, 1988 and 1995.

[20] Boyle P., Parkin D.M. (1991). Cancer Registration: Principles and Methods.

[21] United States Census Bureau (2019). 2010–2018 National and State Population Estimates. https://www.cdc.gov/nchs/nvss/bridged_race.htm.

[22] United States Census Bureau (2022). National Intercensal Datasets 2000–2010. https://seer.cancer.gov/popdata/.

[23] Dasbach E.J., Insinga R.P., Elbasha E.H. (2008). The epidemiological and economic impact of a quadrivalent human papillomavirus vaccine (6/11/16/18) in the UK. BJOG.

[24] Gamerman D., Lopes H.F. (2006). Markov Chain Monte Carlo: Stochastic Simulation for Bayesian Inference.

[25] Giuliano A.R., Viscidi R., Torres B.N., Ingles D.J., Sudenga S.L., Villa L.L., Baggio M.L., Abrahamsen M., Quiterio M., Salmeron J. (2015). Seroconversion following anal and genital HPV infection in men: The HIM study. Papillomavirus Res..

[26] Goodman M.T., Shvetsov Y.B., McDuffie K., Wilkens L.R., Zhu X., Thompson P.J., Ning L., Killeen J., Kamemoto L., Hernandez B.Y. (2008). Prevalence, acquisition, and clearance of cervical human papillomavirus infection among women with normal cytology: Hawaii Human Papillomavirus Cohort Study. Cancer Res..

[27] Hethcote H.W. (1997). An age-structured model for pertussis transmission. Math. Biosci..

[28] Ingles D.J., Lin H.Y., Fulp W.J., Sudenga S.L., Lu B., Schabath M.B., Papenfuss M.R., Abrahamsen M.E., Salmeron J., Villa L.L. (2015). An analysis of HPV infection incidence and clearance by genotype and age in men: The HPV Infection in Men (HIM) Study. Papillomavirus Res..

[29] Kung H.C., Hoyert D.L., Xu J., Murphy S.L. (2008). Deaths: Final Data for 2005. https://pubmed.ncbi.nlm.nih.gov/18512336/.

[30] Laumann E.O., Gagnon J.H., Michael R.T., Michaels S. (2000). The Social Organization of Sexuality: Sexual Practices in the United States.

[31] Lewis R.M., Markowitz L.E. (2018). Human papillomavirus vaccination coverage among females and males, National Health and Nutrition Examination Survey, United States, 2007–2016. Vaccine.

[32] Martinez G.M., Daniels K., Febo-Vazquez I. (2018). Fertility of Men and Women Aged 15–44 in the United States: National Survey of Family Growth, 2011–2015. Natl. Health Stat. Rep..

[33] Mosher W.D., Chandra A., Jones J. (2005). Sexual Behavior and Selected Health Measures: Men and Women 15–44 Years of Age, United States, 2002. https://pubmed.ncbi.nlm.nih.gov/16250464/.

[34] Syrjänen S., Waterboer T., Rintala M., Pawlita M., Syrjänen K., Louvanto K., Grenman S. (2022). Maternal HPV-antibodies and seroconversion to HPV in children during the first 3 years of life. Sci. Rep..

[35] Dunne E.F., Sternberg M., Markowitz L.E., McQuillan G., Swan D., Patel S., Unger E.R. (2011). Human papillomavirus (HPV) 6, 11, 16, and 18 prevalence among females in the United States - National Health and Nutrition Examination Survey, 2003–2006: Opportunity to measure HPV vaccine impact?. J. Infect. Dis..

[36] Liu G., Markowitz L.E., Hariri S., Panicker G., Unger E.R. (2016). Seroprevalence of 9 human papillomavirus types in the United States, 2005–2006. J. Infect. Dis..

[37] Omland T., Akre H., Lie K.A., Jebsen P., Sandvik L., Brøndbo K. (2014). Risk factors for aggressive recurrent respiratory papillomatosis in adults and juveniles. PLoS ONE.

[38] Rock K.S., Huang C.I., Crump R.E., Bessell P.R., Brown P.E., Tirados I., Solano P., Antillon M., Picado A., Mbainda S. (2022). Update of transmission modelling and projections of gambiense human African trypanosomiasis in the Mandoul focus, Chad. Infect. Dis. Poverty.

[39] Chesson H.W., Forhan S.E., Gottlieb S.L., Markowitz L.E. (2008). The potential health and economic benefits of preventing recurrent respiratory papillomatosis through quadrivalent human papillomavirus vaccination. Vaccine.

[40] Seto K., Marra F., Raymakers A., Marra C.A. (2012). The cost effectiveness of human papillomavirus vaccines: A systematic review. Drugs.

[41] Mahumud R.A., Alam K., Keramat S.A., Ormsby G.M., Dunn J., Gow J. (2020). Cost-effectiveness evaluations of the 9-Valent human papillomavirus (HPV) vaccine: Evidence from a systematic review. PLoS ONE.

[42] Cody P., Tobe K., Abe M., Elbasha E.H. (2021). Public health impact and cost effectiveness of routine and catch-up vaccination of girls and women with a nine-valent HPV vaccine in Japan: A model-based study. BMC Infect. Dis..

[43] Nantel É., Mayrand M.H., Audibert F., Niyibizi J., Brassard P., Laporte L., Lacaille J., Zahreddine M., Fraser W., Francoeur D. (2024). Association between the Mode of Delivery and Vertical Transmission of Human Papillomavirus. Viruses.

[44] Medeiros L.R., Ethur A.B.d.M., Hilgert J.B., Zanini R.R., Berwanger O., Bozzetti M.C., Mylius L.C. (2005). Vertical transmission of the human papillomavirus: A systematic quantitative review. Cad. Saude Publica.

[45] Chatzistamatiou K., Sotiriadis A., Agorastos T. (2016). Effect of mode of delivery on vertical human papillomavirus transmission—A meta-analysis. J. Obstet. Gynaecol..

[46] Yan P., Chowell G. (2019). Quantitative Methods for Investigating Infectious Disease Outbreaks.

[47] Gerein V., Rastorguev E., Gerein J., Draf W., Schirren J. (2005). Incidence, age at onset, and potential reasons of malignant transformation in recurrent respiratory papillomatosis patients: 20 years experience. Otolaryngol.-Head Neck Surg..

[48] Brisson M., Bénard É., Drolet M., Bogaards J.A., Baussano I., Vänskä S., Jit M., Boily M.C., Smith M.A., Berkhof J. (2016). Population-level impact, herd immunity, and elimination after human papillomavirus vaccination: A systematic review and meta-analysis of predictions from transmission-dynamic models. Lancet Public Health.

[49] Ryser M.D., Myers E.R., Durrett R. (2015). HPV clearance and the neglected role of stochasticity. PLoS Comput. Biol..

[50] Ryser M.D., Gravitt P.E., Myers E.R. (2017). Mechanistic mathematical models: An underused platform for HPV research. Papillomavirus Res..

[51] Ponduri A., Azmy M.C., Axler E., Lin J., Schwartz R., Chirilă M., Dikkers F.G., Yang C.J., Mehta V., Gangar M. (2023). The efficacy of human papillomavirus vaccination as an adjuvant therapy in recurrent respiratory papillomatosis. Laryngoscope.

