# Supplementary material for: Forecasting Disease Burden with a Dynamic Transmission Model of Human Papillomavirus and Recurrent Respiratory Papillomatosis in the United States
Source: Viruses. 2024 Aug 11;16(8):1283. doi: 10.3390/v16081283 (PMC11359546; doi:10.3390/v16081283)
Supplement: Supplementary file 1 [file viruses-16-01283-s001.zip › viruses-3112046-supplementary.pdf]

Contents

1 Model Description and Static Parameters 1
1.1 The Demographic Model 1
1.1.1 Age Groups 2
1.1.2 All-Cause Mortality and Population size 2
1.1.3 Sexual Behavior 3
1.1.4 Age of Mother at Birth 4
1.2 The Epidemiological Model 4
1.2.1 HPV Transmission and RRP Progression/Clearance 4
1.2.2 HPV Vaccination 7
1.3 JORRP Cohort Model 10
1.4 The Economic Model: Costs and Quality of Life 10
2 Model Calibration 11
2.1 Data 11
2.1.1 HPV Infection Data 11
2.1.2 RRP Data 12
2.2 Calibration Methodology 12
2.3 Calibration Results 15
2.3.1 Fits and Calibration Diagnostics 15
2.3.2 Posterior Distributions 15
3 Bibliography 26

1 Model Description and Static Parameters

Our model for recurrent respiratory papillomatosis (RRP) is based on a model that has been used in several other health economic assessments for human papillomavirus (HPV) vaccination in the United States (US) and other countries [1-6]. It consists of three main parts, a demographic model which reproduces the age and sexual activity structure of the population, an epidemiological model for the transmission of HPV, HPV vaccination, and the progression to disease. Finally, an economic model, which assesses the costs and quality-of-life associated with various interventions.

1.1 The Demographic Model

The demographic model is a set of differential equations constructed based on all-cause mortality, population size, and distribution of sexual behaviors. The fundamental mathematical principles can be found in the seminal work of Hethcote [7]. The relevant parameters used in these computations are found in the subsequent sections.

1.1.1 Age Groups

The age groups chosen for the model reflect the data that is used to inform the model. Among these are the age of diagnosis for juvenile-onset RRP (JORRP) [8], and the various age groups receiving vaccination [8, 9]. These age groups are as follows:

Lower Age	Upper Age
0	0
1	1
2	2
3	3
4	5
6	8
9	9
10	13
14	15
16	18
19	19
20	24
25	28
29	29
30	34
35	39
40	44
45	49
50	54
55	59
60	64
65	69
70	74
75	79
80	84
85	∞

Table 1: Age groups used in the model

1.1.2 All-Cause Mortality and Population size

All-cause mortality (per 100,000) is given below for 5 year age groups + infants [10]. A US population size of 323,071,342 (from 2016) [11] is assumed.

Age	Male	Female
0-0	762.3	619.4
1-4	33.4	25.1
5-9	15.6	13.4
10-14	21.5	14.4

15-19	91.6	37.2
20-24	143.9	48.2
25-29	138.7	54.4
30-34	148.1	73.7
35-39	189.4	109.3
40-44	292.7	174.7
45-49	443.7	264.6
50-54	666	381.6
55-59	925.7	554.3
60-64	1410	887.9
65-69	2084.2	1364.7
70-74	3267	2164
75-79	510.4	3464.7
80-84	8147.4	5822
85+	14889.4	13297.7

Table 2: All-cause mortality for males and females in the US

1.1.3 Sexual Behavior

There are three levels of sexual activity in the model that are based on the average number of annual partners. Sexual activity is a static characteristic in the model, meaning that there is no transfer between classes of sexual activity, although sexual activity does vary with age. Data based on sexual activity is combined with data of age specific activity. The computations that combine these two dimensions, to produce age and activity specific partnership rates can be found in Elbasha et al (2007) [6].

The values for age-specific partnerships, and activity specific partnerships can be found in Table 3 - Table 5 below. The distribution of the population for the sexual activity, and the average number of partners per category can be found in Mosher et al [12]. The sexual mixing properties can be found in Laumann et al [13].

Category	Males (%)	Females (%)
Low (mean number of sexual partners/year: ≤ 1)	77	87
Medium (mean number of sexual partners/year: 2-4)	19	12
High (mean number of sexual partners/year: 5+)	4	1

Table 3: Percent of the population in each of the sexual activity categories.

Sexual activity category	Males (number)	Females (number)
Low (mean number of sexual partners per year: 0-1)	0.75	0.92
Medium (mean number of sexual partners per year: 2-4)	2.52	2.43
High (mean number of sexual partners per year: 5+)	6.10	7.14

Table 4: Average number of partners by sexual activity category.

Age Group	Males	Females	Source
13*	0.00004	0. 00011	Assumption based on Abma [14]
14-15	0.49	1.36	Mosher [12]
16-18	1.02	1.67	
19	1.20	1.65	
20-24	1.43	1.40	
25-26	1.32	1.16	
27-29	1.19	1.13	
30-34	1.20	1.06	
35-39	1.08	1.02	
40-44	1.09	0.96	
45-49	0.91	0.93	Assumption based on Lauman [13]
50-54	0.001 * 0.85	0.001 * 0.83	
55-59	0.001 * 0.74	0.001 * 0.63	
60-64	0.001 * 0.61	0.001 * 0.61	
65-69	0.001 * 0.61	0.001 * 0.61	
70-74	0.001 * 0.44	0.001 * 0.44	
75+**	0.001 * 0.44	0.001 * 0.44	

*Table 5: Sexual partnership rates. *Rates for 13 were obtained by multiplying 14-15 mean by 8% (cumulative percent of never-married male and female persons 15–19 years of age who have ever had sexual intercourse before reaching selected age 14).**Assumption.*

1.1.4 Age of Mother at Birth

Transmission of HPV from mother to child is going to be governed by the prevalence of HPV among child-bearing mothers. As such, it is important for us to accurately capture the distribution of the age of the mother a birth, to properly estimate the risk of transmission, and the impact that interventions may have. We use fertility data that was estimated from the National Survey of Family Growth (NSFG) 2011-2015 [15], and is displayed below in Table 6.

Age Group at Birth	Thousands of Births
15-19	2021
20-24	5053
25-29	5898
30-44	7191

Table 6: Age of Mothers at Birth.

1.2 The Epidemiological Model

The epidemiological model is a compartmental model of HPV transmission and vaccination, as well as progression to RRP. Separate models were constructed for the two types of interest: HPV6 and HPV11. Both models have the same fundamental structure.

1.2.1 HPV Transmission and RRP Progression/Clearance

The susceptible portion of the population interacts with infected groups of the opposite sex through sexual contact. The rates of this sexual contact are computed from the age and activity specific partnership rates [5, 6], and the proportion of these partnerships that result in transmission are

determined by the prevalence of HPV in the sexual partners' demographic and by a sex-specific transmission probability.

To capture the transmission of HPV from mother to child, we assume that a percentage of infants are infected at birth. This percentage is computed by taking a weighted average of the age specific prevalence of HPV among women in the age groups between 15-44, where the weights are derived from the data in 1.1.4. Additionally, as with sexual interactions, this percentage is further reduced by a vertical transmission probability.

HPV infection clears at a sex and age specific rate, and upon clearance, a proportion of individuals will seroconvert, imparting to then some degree of protection against subsequent infection. In our model, this protection does not wane. Seroconversion probabilities and this degree of protection from infection are sex specific.

Progression to RRP is governed by an age specific rate among the HPV infected population and is calibrated against data on the age of diagnosis. Clearance of RRP is computed from data collected from a patient cohort out of Norway [16], where we have assumed that observation time is a proxy for RRP duration. The patient cohort was divided into JORRP and AORRP and the median and IQR of the observation time was given for each. From this distribution and an assumption of a constant hazard for clearance, we compute a likelihood that, given a particular clearance rate γ_{rrp}, the data's distribution would be observed:

$$\mathrm{l}\left((Q_{.25}, Q_{.5}, Q_{.75})|\gamma_{rrp}\right) = Binom\left(n/4; n, 1 - exp\left(-\gamma_{rrp} \cdot Q_{.25}\right)\right)Binom\left(n/2; n, 1 - exp\left(-\gamma_{rrp} \cdot Q_{.5}\right)\right)Binom\left(3n/4; n, 1 - exp\left(-\gamma_{rrp} \cdot Q_{.75}\right)\right)$$

Where $Binom(m; n, p)$ is the probability mass function for a binomial random variable with parameters n, p. Q_p is the quartile for a proportion p and is given by the data. n is the size of the patient cohort in the data. The likelihood l was maximized, and the results are given in Table 7. In principle, we are finding the optimal clearance rate γ_{rrp} such that the cumulative distribution function of the corresponding exponential distribution best fits the observed data. Plots of these fits are given in Figure 1 and Figure 2. RRP clearance rate is assumed to be independent of HPV type.

	n	$Q_{.25}$ (years)	$Q_{.5}$ (years)	$Q_{.75}$ (years)	Fitted Value of γ_{rrp}
JORRP	50	3.7	12.9	32.9	0.0466
AORRP	174	0.8	4.0	11.7	0.1521

Table 7: Data and fitted values for computing RRP clearance.

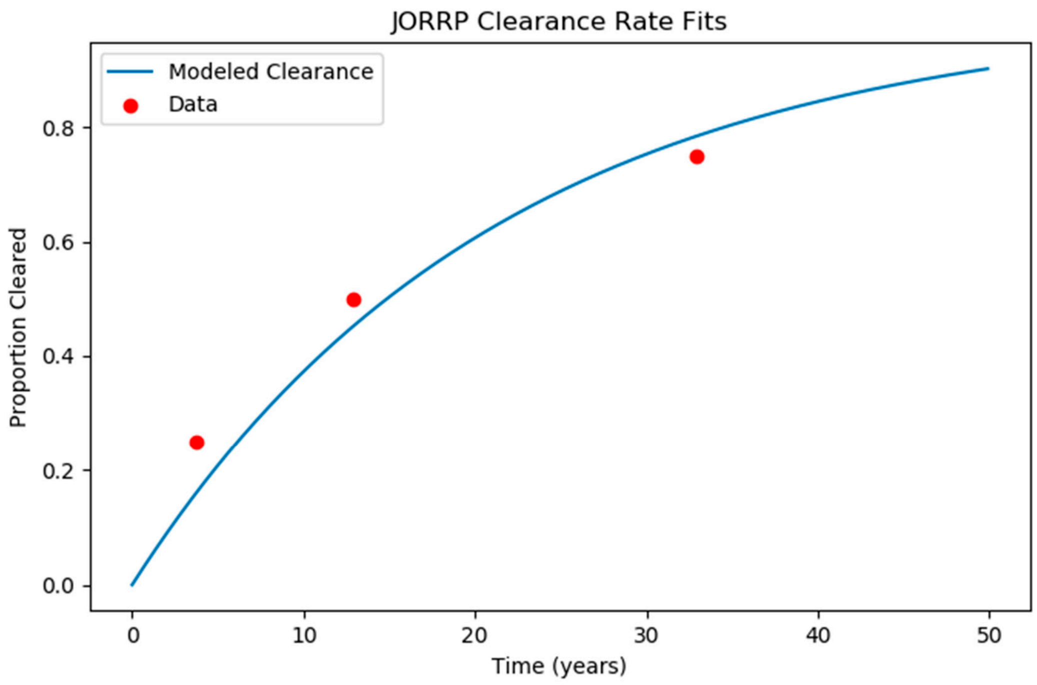

Figure 1: Fits for JORRP clearance against data. As mentioned in the text, observation time in the patient cohort is taken as a proxy for RRP clearance.

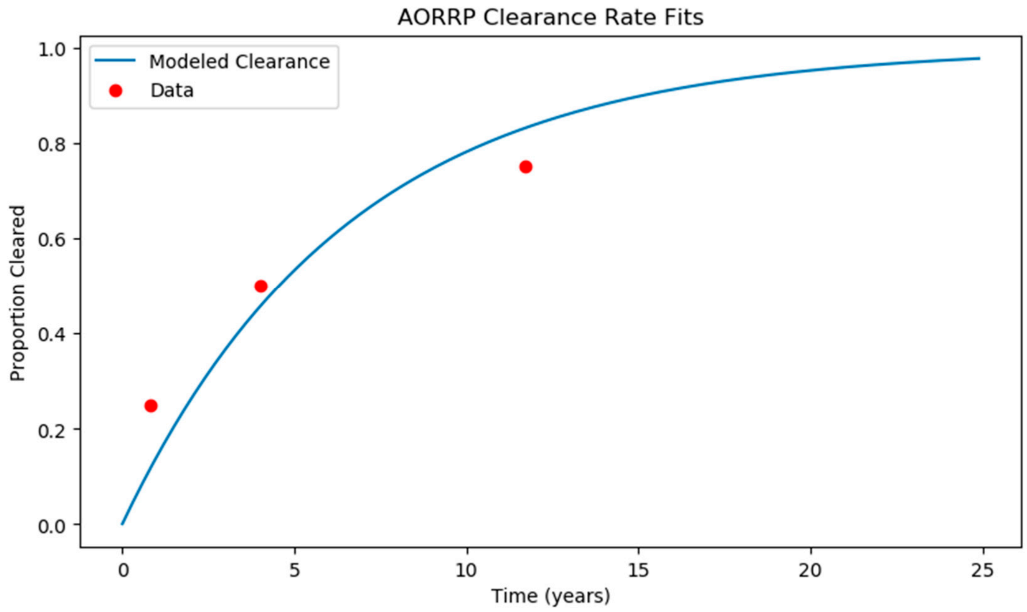

Figure 2: Fits for AORRP clearance against data. As mentioned in the text, observation time in the patient cohort is taken as a proxy for RRP clearance.

RRP mortality is based on estimates from prevalent cases and annual deaths: 4 deaths per 254 to 763 prevalent cases [17]. Hence the range of the probability of death is .00524 to .001572. We take as an estimate (as was done in [17]) that .01 is the annual probability of death for people with RRP. We recognize as a limitation that this mortality is for JORRP, and we have extrapolated it to AORRP.

Most of the parameters involved in HPV transmission were calibrated, utilizing data and prior ranges from the literature, as will be described in later sections.

1.2.2 HPV Vaccination

HPV vaccination occurs at an age specific rate and is computed to match with data gleaned from NIS-TEEN and NHANES.

Data on vaccine coverage for at least one dose of 4vHPV for the ages 13-17 from 2007 to 2019 were obtained from NIS-TEEN [18]. This data was presented for individual age groups in yearly cycles and is presented below in Table 8 and Table 9. For ages 18 and over, we used data from NHANES [9] that gave vaccination coverage data in two-year cycles for age groups, as opposed to individual ages. This data is reproduced below in Table 10 and Table 11.

As can be seen from Table 1, the age groups used in the model do not perfectly mesh with those given in the vaccine coverage data. For ages 9-17, the data from NIS-TEEN is group into weighted averages of the model age groups, whose weights are based on population sizes gleaned from the National Intercensal Datasets for 2000 through 2010 [19]. The NHANES data contained data for ages 9-17, but this was not included into the data. The NHANES data was used to populate vaccine coverage for age groups 20-34, with over 35 years of age being neglected due to low coverage. Vaccine coverage in the model is on an annual basis, so the coverage in the two-year cycle of NHANES was imputed to both years in the model. Since the NHANES data only goes up to 2016, in our baseline scenarios, we assume that no vaccination occurs in these age groups after 2016, because the coverage in primary cohorts will be catching up to these age groups.

Data from Table 8 - Table 11 is integrated into the model through a piecewise constant vaccination rate for sex k in age group i, denoted by $\phi_{k,i}(t)$, where the rate is chosen so that the vaccine coverage achieved in the age group matches the age-group-adjusted data. This is accomplished through a linear vaccination sub-model, where a system of age-stratified ODEs with unvaccinated and vaccinated compartments is optimized and solved sequentially (by age) to achieve the desired coverage. More details on this process can be found in [2].

The age-group-adjusted VCR data, and vaccination rates $\phi_{k,i}(t)$ can be found in Table 12 - Table 15.

Age	2007	2008	2009	2010	2011	2012	2013	2014	2015	2016	2017	2018	2019
13	0.258	0.352	0.371	0.389	0.416	0.468	0.506	0.511	0.564	0.547	0.645	0.611	0.684
14	0.228	0.338	0.406	0.485	0.455	0.494	0.551	0.566	0.612	0.627	0.678	0.686	0.751
15	0.274	0.422	0.46	0.511	0.564	0.539	0.588	0.61	0.627	0.684	0.672	0.707	0.756
16	0.244	0.357	0.499	0.517	0.592	0.558	0.6	0.644	0.63	0.668	0.715	0.735	0.719
17	0.25	0.385	0.471	0.531	0.628	0.642	0.623	0.665	0.706	0.727	0.72	0.763	0.732

Table 8: NIS-TEEN coverage data for at least one does of 4vHPV of 9vHPV vaccine in females.

Age	2007-2010	2011	2012	2013	2014	2015	2016	2017	2018	2019
13	0	0.098	0.195	0.335	0.389	0.487	0.524	0.571	0.64	0.654
14	0	0.082	0.222	0.351	0.426	0.47	0.56	0.624	0.651	0.722
15	0	0.074	0.209	0.362	0.457	0.514	0.554	0.657	0.687	0.689
16	0	0.098	0.212	0.359	0.4	0.515	0.573	0.634	0.692	0.704
17	0	0.062	0.203	0.321	0.418	0.504	0.586	0.643	0.647	0.716

Table 9: NIS-TEEN coverage data for at least on dose of 4vHPV or 9vHPV vaccine in males.

Age	2007-2008	2009-2010	2011-2012	2013-2014	2015-2016
9-13	11	14.8	22.7	17.5	22.4
14-19	21.1	45.2	54.3	52.4	54.7
20-24	11.8	24.4	39.6	45	56
25-29	5.1	11	19.9	28.8	37.1
30-34	5.3	2.4	3.4	10.4	17.4
35-39	3.1	2.2	0.6	3.7	5.6
40-59	0.5	1.6	1.6	2.3	1.6

Table 10: NHANES coverage data for at least one dose of 4vHPV or 9vHPV vaccine in females.

Age	2007-2008	2009-2010	2011-2012	2013-2014	2015-2016
9-13	0	0	10.4	18.4	24.2
14-19	0	0	8.6	28.2	39.5
20-24	0	0	6.6	14.4	22.6
25-29	0	0	5.2	7.2	12.3
30-34	0	0	2.7	1.2	2.9
35-39	0	0	2.1	3.5	1.8
40-59	0	0	0.1	0.8	1.1

Table 11: NHANES coverage data for at least one dose of 4vHPV or 9vHPV vaccine in males.

Age	2007	2008	2009	2010	2011	2012	2013	2014	2015	2016	2017	2018	2019
10-13	0.06	0.09	0.09	0.10	0.10	0.12	0.13	0.13	0.14	0.14	0.16	0.15	0.17
14-15	0.25	0.38	0.43	0.50	0.51	0.52	0.57	0.59	0.62	0.66	0.67	0.70	0.75
16-18	0.16	0.33	0.45	0.51	0.58	0.61	0.62	0.64	0.67	0.70	0.72	0.74	0.74
19-19	0.00	0.00	0.00	0.00	0.00	0.00	0.00	0.00	0.00	0.00	0.00	0.00	0.00
20-24	0.12	0.12	0.24	0.24	0.40	0.40	0.45	0.45	0.56	0.56	0.00	0.00	0.00

25-28	0.05	0.05	0.11	0.11	0.20	0.20	0.29	0.29	0.37	0.37	0.00	0.00	0.00
29-29	0.00	0.00	0.00	0.00	0.00	0.00	0.00	0.00	0.00	0.00	0.00	0.00	0.00
30-34	0.05	0.05	0.05	0.02	0.03	0.03	0.10	0.10	0.17	0.17	0.00	0.00	0.00

Table 12: Age-group-adjusted target vaccination coverage data for females. A 0 here does not necessarily mean that the target coverage is 0, rather that no active vaccination is taking place in that age group. Some age-groups consisting of a single year have no active vaccination, since the coverage for that group was aggregated into an adjacent age group due to data constraints.

Age	2007-10	2011	2012	2013	2014	2015	2016	2017	2018	2019
10-13	0.00	0.02	0.05	0.08	0.10	0.12	0.13	0.14	0.16	0.16
14-15	0.00	0.08	0.22	0.36	0.44	0.49	0.56	0.64	0.67	0.71
16-18	0.00	0.05	0.16	0.29	0.38	0.48	0.55	0.62	0.66	0.69
19-19	0.00	0.00	0.00	0.00	0.00	0.00	0.00	0.00	0.00	0.00
20-24	0.00	0.07	0.07	0.14	0.14	0.23	0.23	0.00	0.00	0.00
25-28	0.00	0.05	0.05	0.07	0.07	0.12	0.12	0.00	0.00	0.00
29-29	0.00	0.00	0.00	0.00	0.00	0.00	0.00	0.00	0.00	0.00
30-34	0.00	0.03	0.03	0.01	0.01	0.03	0.03	0.00	0.00	0.00

Table 13: Age-group-adjusted target vaccination coverage data for males. A 0 here does not necessarily mean that the target coverage is 0, rather that no active vaccination is taking place in that age group. Some age-groups consisting of a single year have no active vaccination, since the coverage for that group was aggregated into an adjacent age group due to data constraints

Age	2007	2008	2009	2010	2011	2012	2013	2014	2015	2016	2017	2018	2019
10-13	0.08	0.05	0.03	0.03	0.04	0.05	0.05	0.04	0.05	0.04	0.07	0.04	0.07
14-15	0.35	0.37	0.36	0.48	0.43	0.43	0.59	0.59	0.68	0.80	0.83	0.92	1.28
16-18	0.16	0.19	0.19	0.11	0.20	0.14	0.09	0.11	0.12	0.15	0.12	0.12	0.00
19-19	0.00	0.00	0.00	0.00	0.00	0.00	0.00	0.00	0.00	0.00	0.00	0.00	0.00
20-24	0.13	0.00	0.13	0.00	0.15	0.00	0.00	0.00	0.12	0.00	0.00	0.00	0.00
25-28	0.04	0.00	0.02	0.00	0.02	0.00	0.00	0.00	0.00	0.00	0.00	0.00	0.00
29-29	0.00	0.00	0.00	0.00	0.00	0.00	0.00	0.00	0.00	0.00	0.00	0.00	0.00
30-34	0.06	0.00	0.00	0.00	0.00	0.00	0.00	0.00	0.00	0.00	0.00	0.00	0.00

Table 14: The piecewise values of $\phi_{f,i}(t)$ (or force of vaccination) for females in various years and age groups.

Age	2007-10	2011	2012	2013	2014	2015	2016	2017	2018	2019
10-13	0.00	0.03	0.03	0.06	0.04	0.06	0.05	0.05	0.06	0.05
14-15	0.00	0.10	0.23	0.36	0.41	0.44	0.57	0.81	0.82	0.98
16-18	0.00	0.05	0.10	0.15	0.10	0.15	0.15	0.15	0.10	0.07
19-19	0.00	0.00	0.00	0.00	0.00	0.00	0.00	0.00	0.00	0.00
20-24	0.00	0.07	0.00	0.08	0.00	0.05	0.00	0.00	0.00	0.00
25-28	0.00	0.05	0.00	0.01	0.00	0.01	0.00	0.00	0.00	0.00
29-29	0.00	0.00	0.00	0.00	0.00	0.00	0.00	0.00	0.00	0.00
30-34	0.00	0.03	0.00	0.00	0.00	0.00	0.00	0.00	0.00	0.00

Table 15: The piecewise values of $\phi_{m,i}(t)$ (or force of vaccination) for males in various years and age groups.

1.3 JORRP Cohort Model

Since incidence by birth-cohort is a key piece of data that we were using as a target, construction of a cohort model for JORRP was necessary. Construction is perhaps a strong term, since the JORRP cohort model was really a sub-model of the larger model, that is initialized with data from the larger model.

The JORRP cohort model is a compartmental model consisting of the first 8 age groups given in Table 1, separated by gender. Epidemiologically, there are three kinds of compartments in the model: Infected, JORRP, and recovered. The model is initialized with all age groups set at zero, except for the youngest infected compartments. These are initialized to the value of the number of infants, out of a cohort of 100,000, that we expect to be born infected (this value is passed into the cohort model by the larger model). Each of the infected compartments can either clear or progress to RRP. RRP rates of progression are age-specific, but clearance rates of infection are not age-specific. This is done because we did not use any data on age specific prevalence of HPV among juveniles, but age-specific data was used for RRP progression.

One the model is initialized, it is run for 20 years (to reach a steady state), and the number of JORRP cases and their age of progression (or diagnosis) is recorded for comparison with the data.

1.4 The Economic Model: Costs and Quality of Life

The total number of surgeries incurred by the RRP population in computed through integration:

$$N_s(t) = \sum_{i=1}^{age} \sum_{l=1}^{activity} S_i \cdot a_i \int_0^t rrpf_{l,i}(s) + rrpm_{l,i}(s)\, ds$$

Where $N_s(t)$ is the cumulative number of surgeries up to time t, S_i is the median number of surgeries per year among people with RRP in age group i, a_i is the width of the age group, df_i and dm_i are the aging rates for males and females, and $rrpf_i(s)$ and $rrpm_i(s)$ are the number of individuals with RRP in age group i. Actual costs, $C(t)$, are computed in a similar way, letting p be the price of a single surgery:

$$C(t) = \sum_{i=1}^{age} \sum_{l=1}^{activity} S_i \cdot p \cdot a_i \int_0^t exp(-\zeta s)\left(rrpf_{l,i}(s) + rrpm_{l,i}(s)\right) ds$$

ζ is based on the discount rate. For example, for a discount rate of 3%, we let $\zeta = ln(1 + .03)$.

The median number of surgeries is type specific and age specific, with JORRP caused by HPV6 incurring 2 surgeries per year, while HPV11-caused JORRP incurs 3 surgeries per year [20]. For AORRP we used an estimate of 1.2 surgeries per year [16]. The cost per surgery is based on a 2000 study [17], which estimated $4817 per surgery. Depending on the context, this price in inflated to match the appropriate date e.g., when computing retrospective costs, it is inflated to the appropriate year.

Quality of life is measured through Quality Adjusted Life Years (QALYs). In the model, the QALYs lost due to morbidity of RRP are computed by integrating the RRP states over the desired time interval, multiplied by an age-specific utility, and an RRP utility of $.74/.93 = .7957$ [2], and may be discounted. The same utility is used for both JORRP and AORRP. For sensitivity, we use a standard deviation of .15, and thus a Beta distribution with parameters 5.4789 and 1.4761. The

subpopulation that dies from RRP moves to a separate compartment, and the QALYs lost due to death are estimated by integrating these death states with the standard age-specific utility, discounted appropriately.

2 Model Calibration

For a rare disease like RRP, there is substantial uncertainty around quantitative aspects of the disease. To calibrate the model in a way that accounts for this uncertainty we relied on Bayesian inference to derive posterior distributions for various parameters (See the excellent text of Gamerman et al [21] for an introduction). First, we will describe the data used to calibrate the model. Then we will lay out the process by which the posterior distributions were derived. Finally, we will show the posterior distributions and the model fits.

2.1 Data

Broadly, the data used to calibrate the model can be classed into two groups: HPV infection data, and RRP data. HPV infection represents an important target to hit, since that is the only way to correctly capture the risk of infection for newborn infants. All data that the model was calibrated to represents pre-HPV-vaccination targets (2007), and we assumed that it represented a steady state for the dynamics before the vaccine intervention was introduced.

2.1.1 HPV Infection Data

Our HPV infection targets for females were HPV prevalence data analyzed from NHANES [22]. This data, along with its confidence intervals in presented below in Table 16.

HPV infection data for male was represented by seroprevalence data, again analyzed from NHANES [23]. HPV types 6 and 11 were aggregated for the age specific seroprevalence but were reported separately for overall seroprevalence. Overall, among males, the seroprevalence was 9.4% and 3.0% for HPV6 and HPV11 respectively. To extrapolate to the age-specific data we assumed that the distribution of type 6 to type 11 cases was the same for each age group i.e. (9.4/(9.4+3)) = .7581 is the proportion of seroprevalence due to HPV6, and (3/(9.4+3)) = .2419 is the proportion of seroprevalence due to HPV11. The seroprevalence targets that emerge from this calculation are given below in Table 17. This seroprevalence ratio has its own uncertainty that we did not consider. As such we are likely underestimating the uncertainty in male seroprevalence.

Age Group	Prevalence HPV6	Prevalence HPV11
14-19	0.054 (0.038, 0.076)	0.01 (0.005,0.022)
20-24	0.037 (0.024, 0.058)	0.004 (0.001, 0.033)
25-29	0.04 (0.024, 0.066)	0 (0, 0.007)
30-39	0.031 (0.019, 0.049)	0.002 (0, 0.007)
40-49	0.014 (0.006, 0.031)	0.004 (0.001, 0.021)
50-59	0.017 (0.008, 0.036)	0.002 (0, 0.013)

Table 16: Prevalence of HPV6 and HPV11 in females by age

Age Group	HPV6/11 Seroprevalence	HPV6 Seroprevalence Estimate	HPV11 Seroprevalence Estimate
14-19	0.023 (0.012, 0.041)	0.017 (0.009, 0.031)	0.006 (0.003, 0.010)
20-29	0.095 (0.062, 0.143)	0.072 (0.047, 0.108)	0.023 (0.015,0.035)
30-39	0.119 (0.09, 0.155)	0.09 (0.068, 0.118)	0.029 (0.022, 0.038)

40-49	0.137 (0.096, 0.193)	0.104 (0.073, 0.146)	0.033 (0.023, 0.047)
50-59	0.118 (0.074, 0.182)	0.089 (0.056, 0.138)	0.029 (0.018, 0.044)

Table 17: Combined HPV6/11 seroprevalence from NHANES, with our type-specific seroprevalence estimates. All values are rounded to three digits.

2.1.2 RRP Data

For AORRP we used the well-known incidence estimate of 1.8 per 100,000 [24].

For JORRP incidence we used the very recent study of Meites et al [8] that gave an estimates of risk among birth cohorts. We took the incidence of the 2004-2005 birth cohort, with national denominator data, of 2 per 100,000 to be the pre-vaccination target for calibration of the model.

Meites et al [8] also collected data on the age of diagnosis of RRP, which was used as a target as well: We assumed that 55.9% of cases were diagnosed before age 4 and between 4 and 8 years of age, 36.6% of cases were diagnosed. The rest were assumed to be diagnosed between the ages of 9 and 13. It is worth noting that 2.6% of the cases in the study did not have a known age of diagnosis. We assumed that these occurred in the last age group. This is conservative relative to economic outcomes since less lifetime surgeries and less loss of quality of life accompany this assumption.

Since separate models were set up for each type, estimates of RRP attribution were needed to generate type-specific targets for calibration. We utilized the recent study of Amiling et al [20], which included HPV typing of JORRP cases. 79.6% of JORRP cases were attributed to HPV6 and 15.4% of JORRP cases were attributed to HPV11. The same attribution was applied to AORRP.

2.2 Calibration Methodology

Calibration was accomplished using the above data, which we will denote as $D = \{d_1, d_2, \dots, d_n\}$ for the sake of convenience, and Bayesian inference on the vector of parameters θ, sampling from the posterior distribution P:

$$P(\theta|D) \propto p(\theta)L(D|\theta).$$

$p(\theta)$ is the prior distribution for the parameters and $L(D|\theta)$ is the likelihood.

The prior distributions are assumed to be uniform, for simplicity, with bounds that are sourced from US estimates for the parameters where available. These prior bounds are provided below in Table 18. The likelihood has the form

$$L(D|\theta) = \prod_{i=1}^{|D|} exp\left(-\frac{(f_i(\theta) - d_i)^2}{\sigma_i}\right)$$

$f_i(\theta)$ is the output from the model corresponding to the data point d_i, and σ_i is the variance of the data point's distribution. The variance for each of the data points is computed in a couple of different ways. The variance for the prevalence and seroprevalence was reconstructed from the confidence intervals. The variance of RRP incidence was computed with standard registry techniques [25].

The functions f_i are unlikely to have a closed analytical form, as they are solutions to a set of nonlinear ODEs. As such, the posterior distribution cannot be identified with any distributions that have an analytical form. This makes the problem of drawing from the posterior amenable to

Metropolis-Hastings MCMC methods since such methods only require us to be able to evaluate the likelihood numerically [21]. Succinctly, a Markov chain whose equilibrium distribution is precisely the posterior distribution is created by proposing a new set of parameters, evaluating the posterior at those new proposed parameters, and accepting them with probability equal to the ratio of the new posterior value and the value of the posterior at the last element (model parameter values) in the chain.

For this problem we utilized an independence chain Metropolis-Hastings algorithm, where the proposal is not based on the previous element of the chain (Note that this does not violate the Markov property of the chain, since whether or not we accept the proposal is based on what the previous element of the chain is). Such chains, while they may have lower acceptance ratios, have less issues with autocorrelations, and achieve good coverage of the prior space. Our proposal distribution was first computed by numerically optimizing the posterior hundreds of times. This produced a set of points that roughly corresponding to peaks in the distribution. We then computed the mean $\bar{\mu}$ and sample covariance $\bar{C}$ of this set of optimal points and defined our proposal distribution as a multivariate normal with mean $\bar{\mu}$ and covariance $\kappa\bar{C}$. κ was a tuned parameter, which was chosen so that we could achieve acceptance rates between 2% and 5%. For the HPV6 model we had $\kappa = 1.1$ and for the HPV11 model we had $\kappa = .75$. However, we did run partial chains with different values of κ to confirm that the distributions did not change.

Parameter (Symbols)	Description	Prior Bounds: HPV6	Prior Bounds: HPV11
Vertical Transmission (♀)	Proportion of infants born to infected mothers who will get an oral HPV infection. Units: None.	$0 < ♀ < 1$<br>Source: Assumption	$0 < ♀ < 1$<br>Source: Assumption
Transmission Probabilities (bf, bm)	Probability that a genital infection will be transmitted during sexual interactions.<br>Units: None.	$0 < bm, bf < 1$<br>Source: Assumption	$0 < bm, bf < 1$<br>Source: Assumption
RRP progression $(jorone, jortwo$<br>$, jorthree, aor)$	The annual rate at which oral infections progress to RRP. Varies by age groups: 0-3, 4-8, 9-13, and 13+.	$0 < jorone < 1$,<br>$0 < jortwo < 2$,<br>$0 < jorthree < 8$,<br>$0 < aor < 0.01$	$0 < jorone < 1$<br>$0 < jortwo < 2$<br>$0 < jorthree < 9$<br>$0 < aor < 0.01$

	Units: $1/year$	Source: Assumption	Source: Assumption
Infant HPV clearance (γpj)	Annual clearance rate of oral HPV infection in children.<br><br>Units: $1/year$	$0 < \gamma pj < 7$<br><br>Source: [26]. Due to the small sample size, the uncertainty was substantial, so we broadened the interval significantly.	$0 < \gamma pj < 7$<br><br>Source: [26]. This study was unable to detect a significant difference in clearance rates of 6 and 11, hence we used the same prior bounds.
Adult HPV clearance (γpm, γpf)	Annual clearance rate of genital HPV infection in adults.<br><br>Units: $1/year$	$0.5 < \gamma pm < 1.5$<br><br>Source: [27]. Converted from duration in months to annual rate.<br><br>$0.5 < \gamma pf < 3.23$<br><br>Source: [28]. Converted median duration in days to an annual rate.	$0.5 < \gamma pm < 1.5$<br><br>Source: [27]. Converted from duration in months to annual rate.<br><br>$0.5 < \gamma pf < 3.23$<br><br>Source: [28]. The small sample size for HPV11 did not allow for a median to be computed. We used the same range as HPV6.
Seroconversion probability (im, if)	Probability that an infected individual seroconverts.<br><br>Units: None.	$0.05 < im < .2$<br><br>Source: [29]. These bounds are derived from the point estimates with standard Central Limit Theorem techniques.<br><br>$0 < if < 1$<br><br>Source: Assumption	$0 < im < .5$<br><br>Source: [29]. These bounds are derived from the point estimates with standard Central Limit Theorem techniques.<br><br>$0 < if < 1$<br><br>Source: Assumption

Degree of Protection (ψzm, ψzf)	Reduction in risk of infection after seroconversion.<br><br>Units: None.	$0 < \psi zm, \psi zf < 1$<br><br>Source: Assumption	$0 < \psi zm, \psi zf < 1$<br><br>Source: Assumption

Table 18: Prior bounds for the calibrated parameters.

2.3 Calibration Results

2.3.1 Fits and Calibration Diagnostics

Fits for HPV6 and HPV11 are provided below in Figure 4 through Figure 8. Generally, the estimated model prevalence and incidence were within acceptable bounds. The exception being among the female genital HPV prevalence among women age 50-59. However, due the small number of women in the age group giving birth, it is unlikely that the failure to accurately model the HPV6 prevalence in this subgroup will impact the results for JORRP. However, if we are underestimating the prevalence of HPV6 in this group, then we are also underestimating the percentage of new AORRP cases that come from infections in this age group, and in the model they will tend to start earlier, which means we could be overestimating the loss of QOL and treatment costs. However, such cases likely represent such a small percentage of AORRP cases, it is unlikely that this underestimate of HPV6 prevalence of women in their 50's has any significant impact on the results.

Diagnostic plots for the Markov chains are given in Figure 9 and Figure 10 .

2.3.2 Posterior Distributions

Summary statistics (means with credibility intervals) are provided in Table 19. In Figure 11 the box plots of the posterior distributions are given by HPV type for comparison.

Parameter (Symbols)	Value: HPV6	Value: HPV11
ϙ	0.8444, (0.5999,1.0000)	0.3855, (0.2040,0.6179)
bm	0.9606, (0.8767,1.0000)	0.8732, (0.6606,1.0000)
bf	0.8267, (0.5906,1.0000)	0.9502, (0.8539,1.0000)
$jorone$	0.0010, (0.0008,0.0014)	0.0054, (0.0032,0.0081)
γpj	2.7120, (2.4590,2.9310)	1.7000, (1.5000,1.9350)

$jortwo$	0.1370, (0.1062,0.1678)	0.2065, (0.1491,0.2660)
$jorthree$	4.8510, (1.6830,8.0000)	4.7380, (1.2920,8.3800)
ψzm	0.2897, (0.0181,0.5464)	0.2789, (0.0000,0.6385)
aor	0.0007, (0.0005,0.0008)	0.0026, (0.0018,0.0035)
γpm	0.9085, (0.6624,1.1750)	1.3410, (0.9508,1.7390)
ψzf	0.8834, (0.7633,0.9914)	0.8641, (0.6542,1.0000)
γpf	1.2850, (0.9238,1.6770)	2.8600, (2.3770,3.2300)
im	0.1422, (0.1216,0.1659)	0.3916, (0.2888,0.5000)
if	0.6038, (0.3331,0.9648)	0.6184, (0.2650,0.9456)

Table 19: Summary statistics for posterior parameter distributions.

Correlations between sampled parameters were assessed, and those with absolute sample correlation ($\hat{\rho}$) greater than 0.7 are plotted below in Figure 12 and Figure 13.

3 Additional Figures

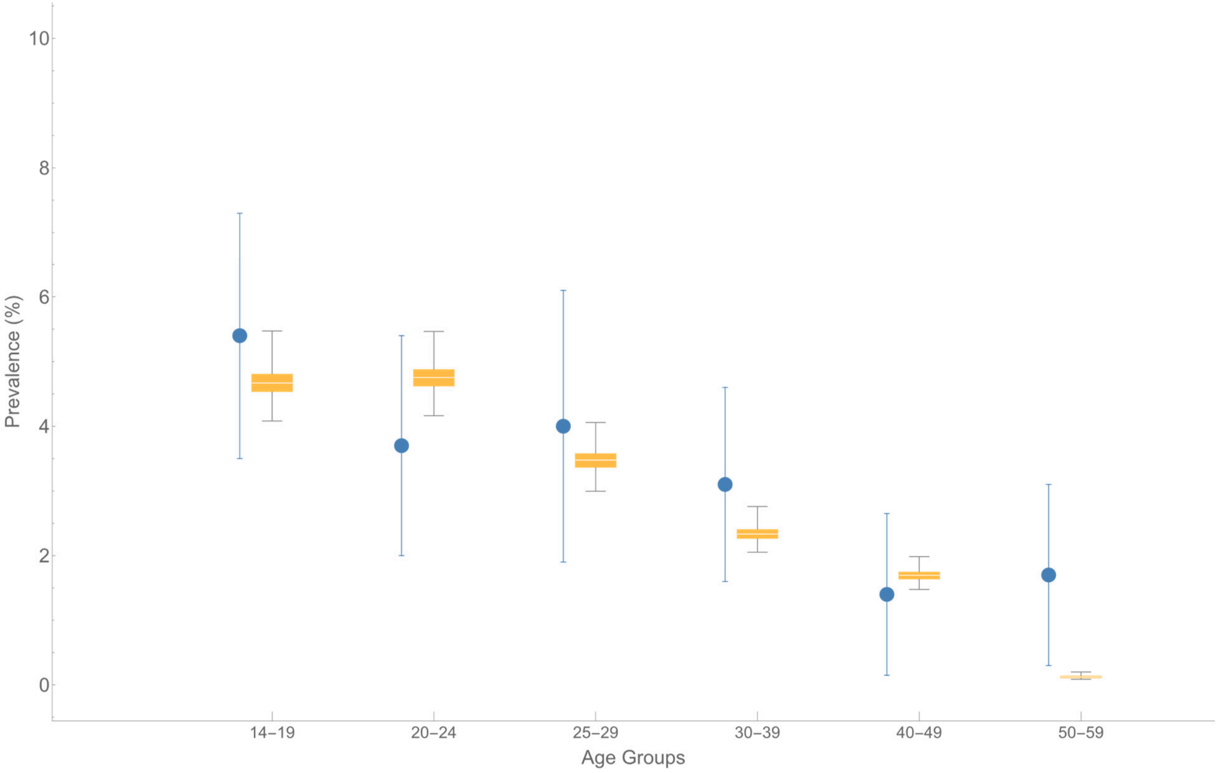

Figure 3: HPV6 model female prevalence fits to data. The yellow boxplots represent the model output, and the blue dots with error bars are the data.

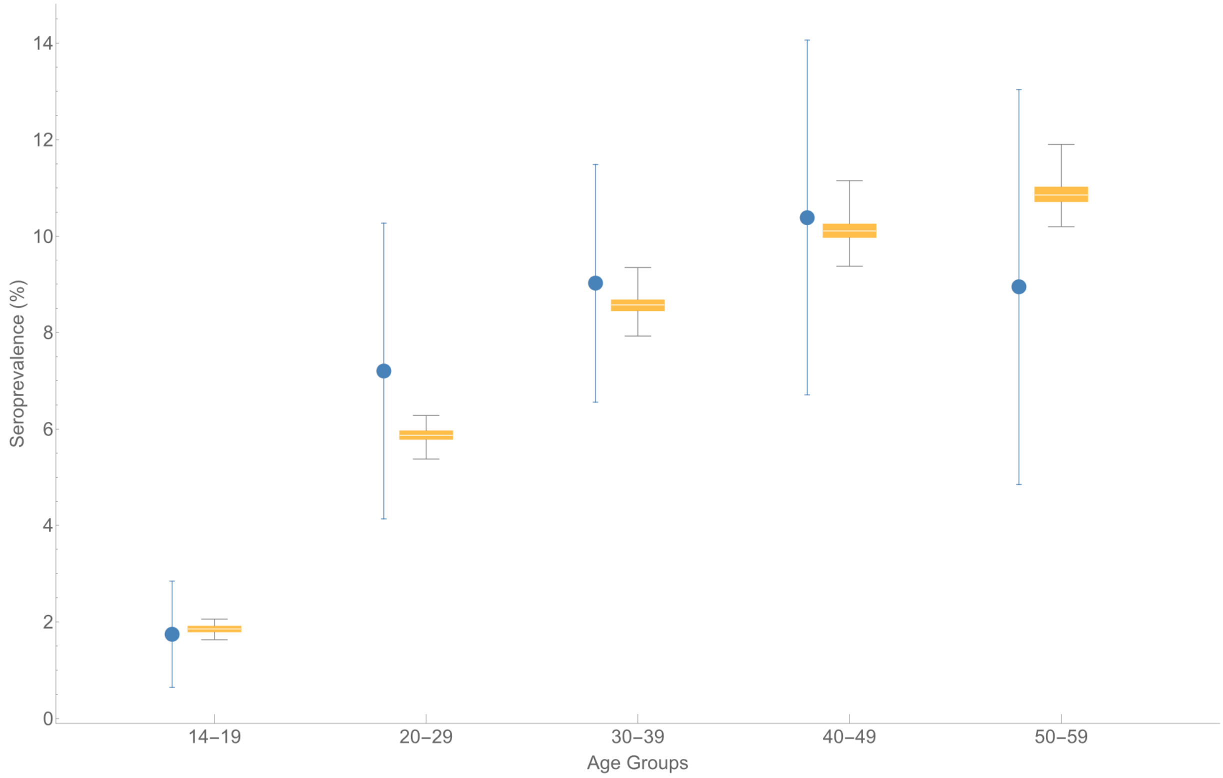

Figure 4: HPV6 model male seroprevalence fits to data. The yellow boxplots represent the model output, and the blue dots with error bars are the data.

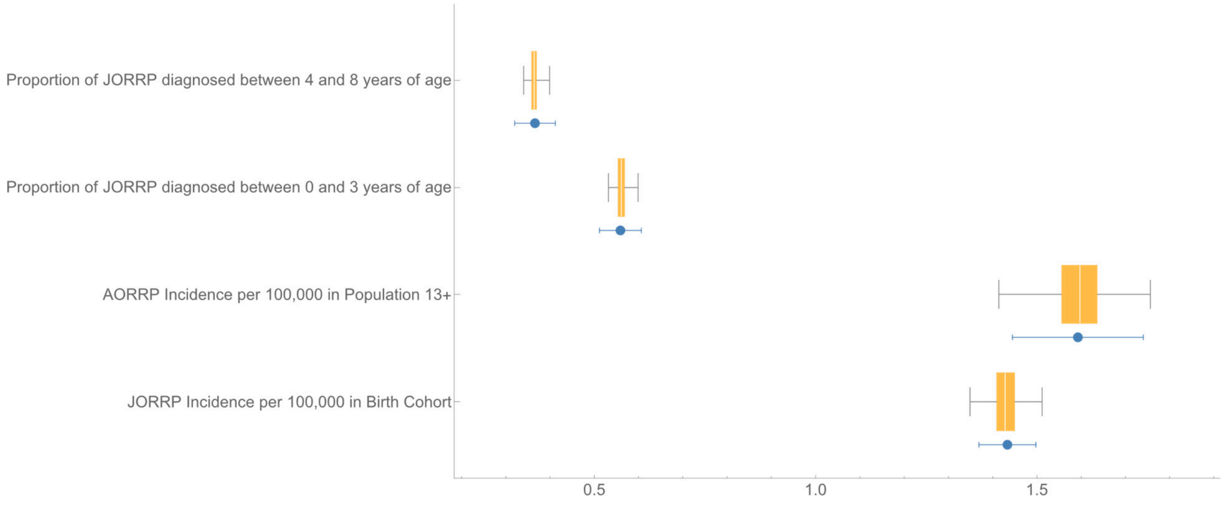

Figure 5: HPV6 model age-of-diagnosis and RRP incidence fits to data. The yellow boxplots represent the model output, and the blue dots with error bars are the data.

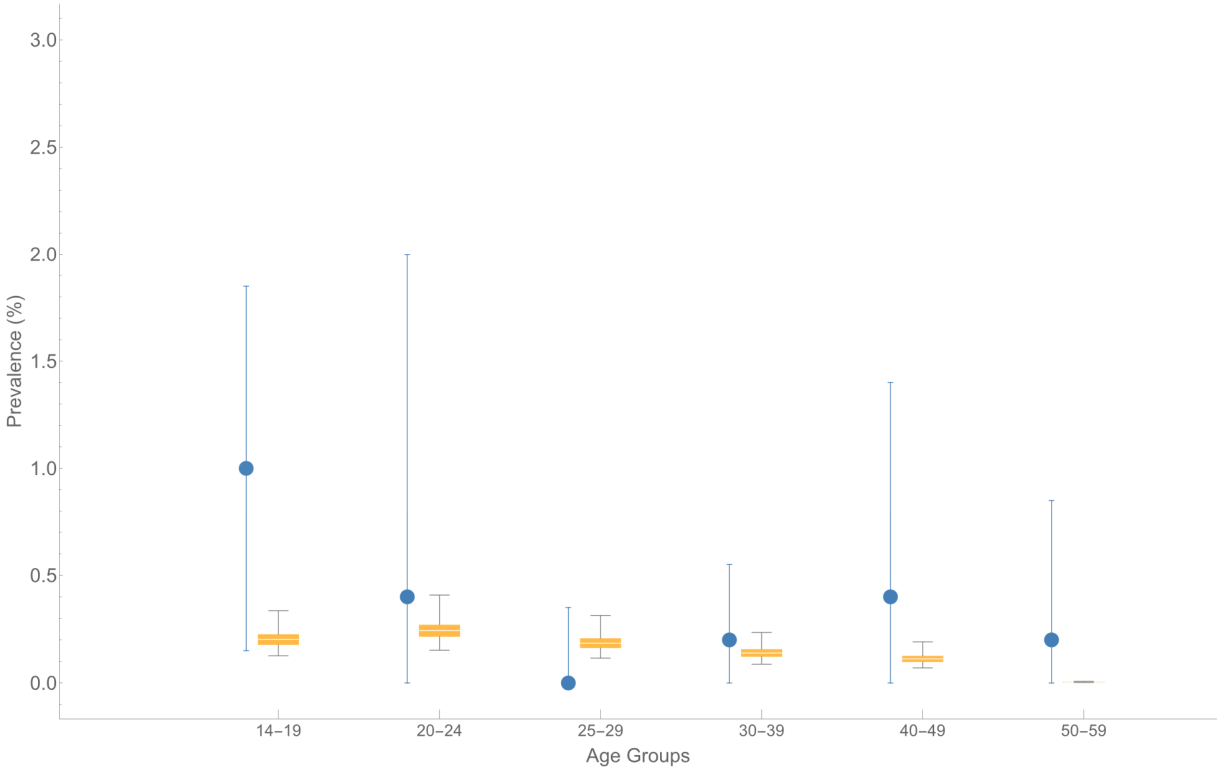

Figure 6: HPV11 model female prevalence fits to data The yellow boxplots represent the model output, and the blue dots with error bars are the data.

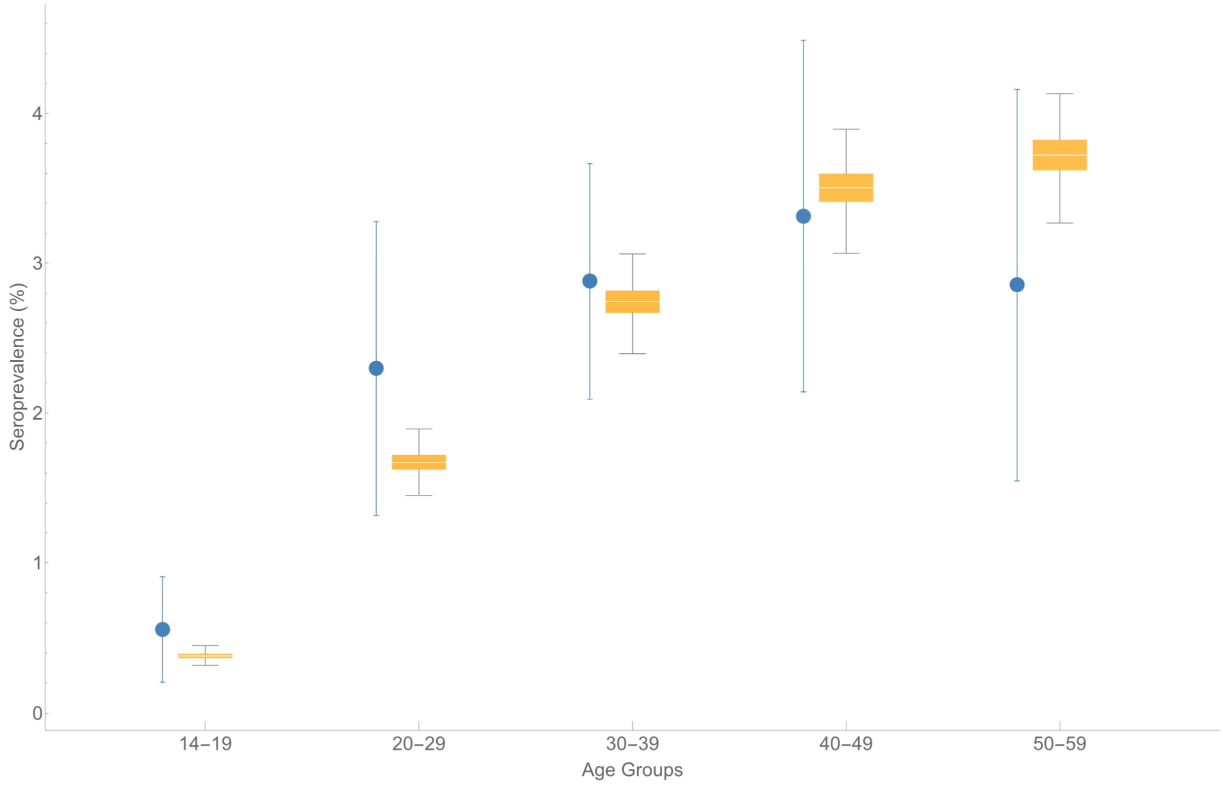

Figure 7:HPV11 model male seroprevalence fits to data. The yellow boxplots represent the model output, and the blue dots with error bars are the data.

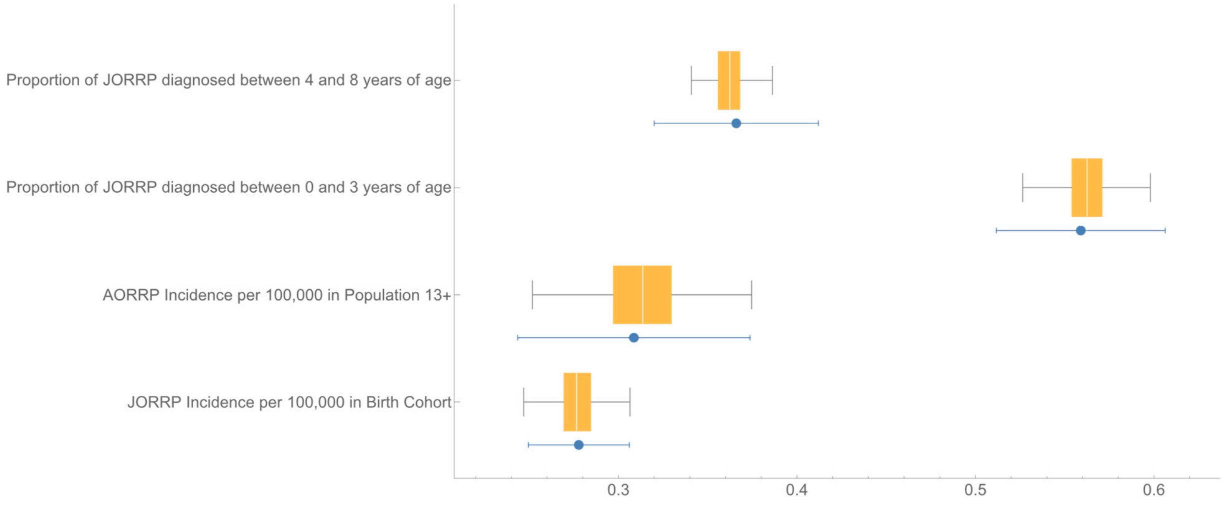

Figure 8: HPV11 model age-of-diagnosis and RRP incidence fits to data. The yellow boxplots represent the model output, and the blue dots with error bars are the data.

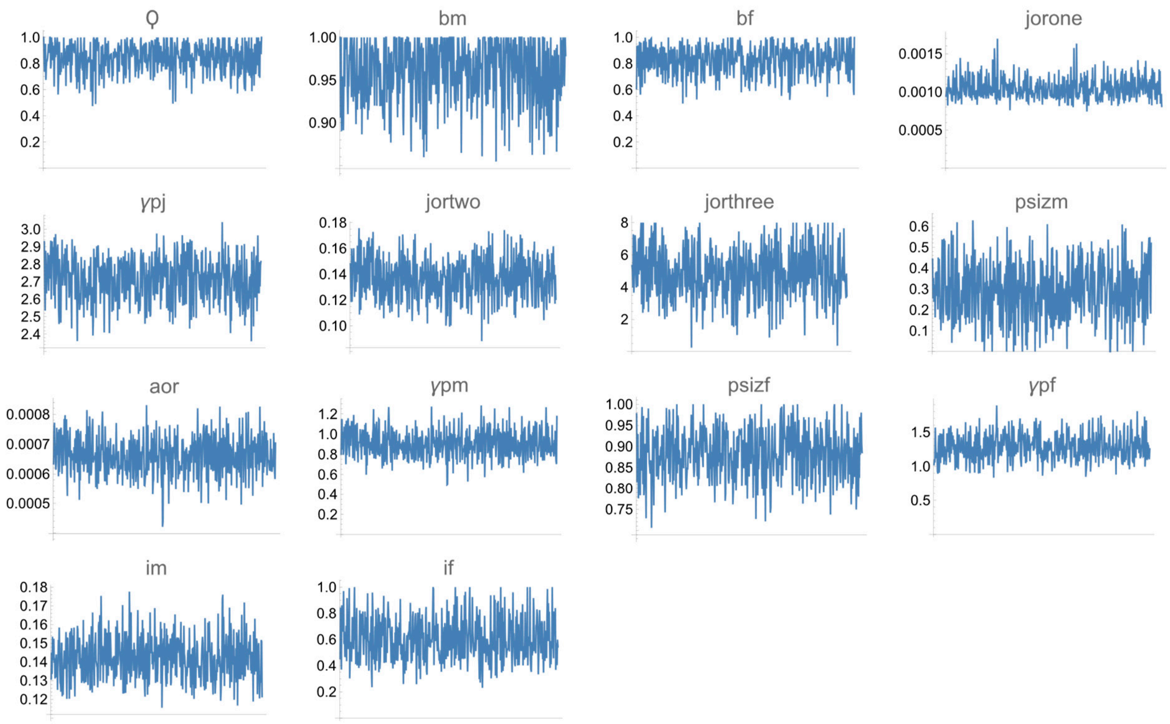

Figure 9: Diagnostic plots for the MCMC sampling for HPV6 model.

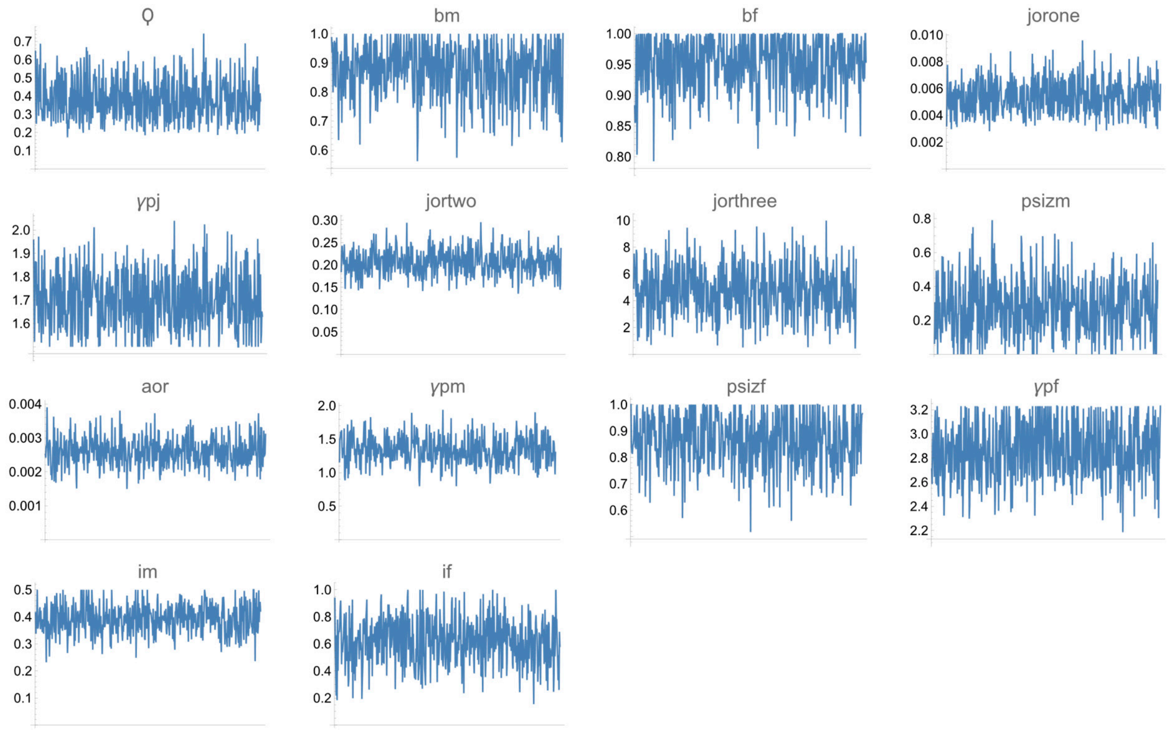

Figure 10: Diagnostic plots for the MCMC sampling for HPV11 model.

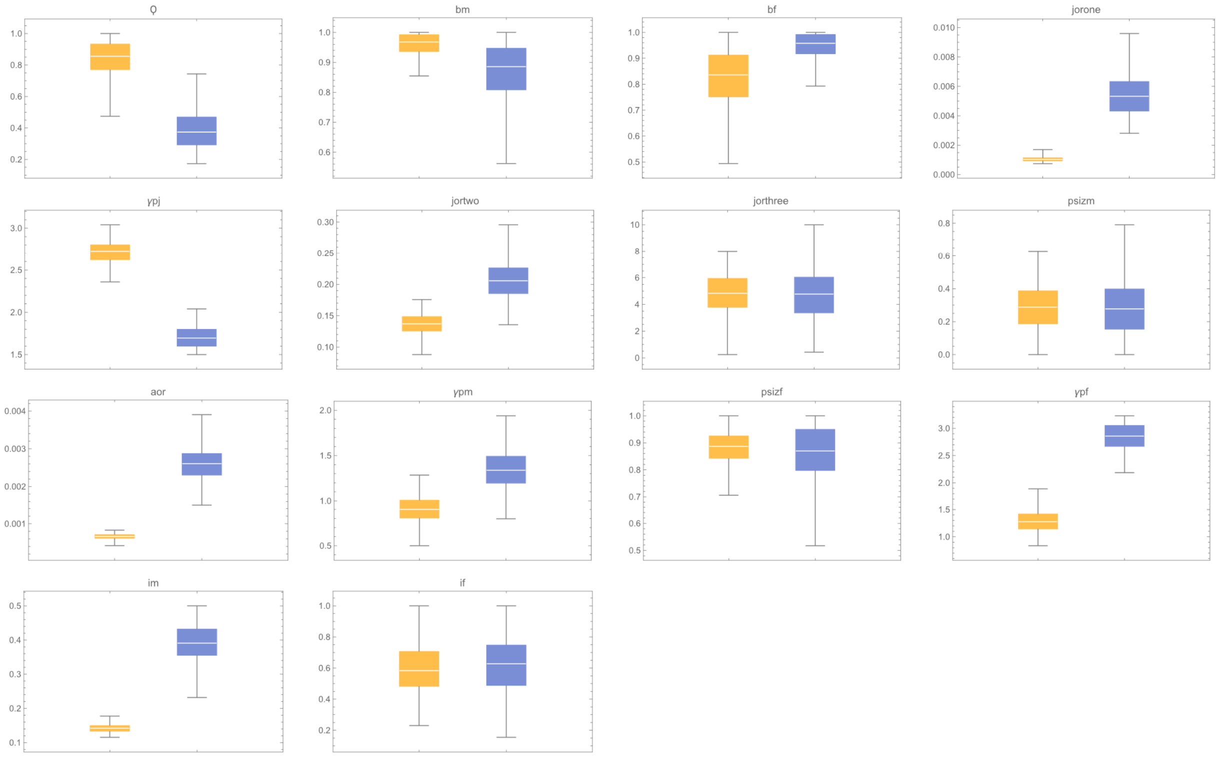

Figure 11: Posterior parameter distributions by HPV type (Yellow is HPV6 and Blue is HPV11). Consult the summary statistics table for definitions of the symbols.

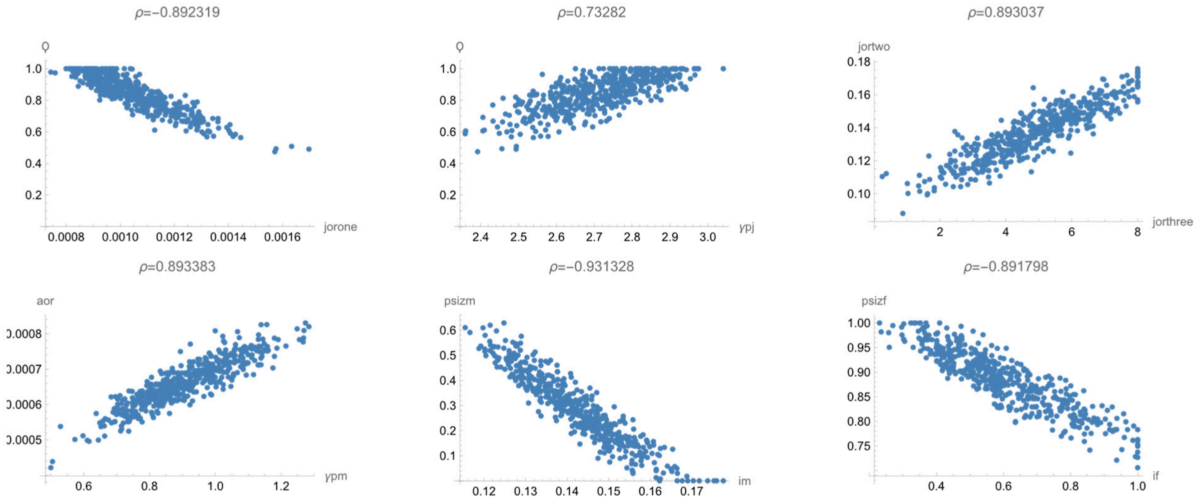

Figure 12: Correlated parameters from the posterior samples for HPV6

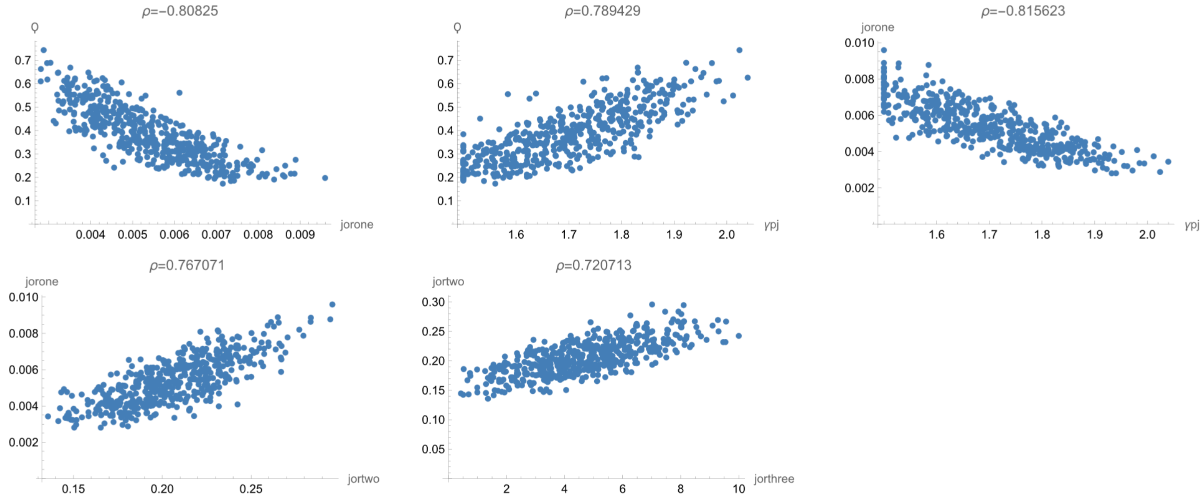

Figure 13: Correlated parameters from the posterior samples for HPV11

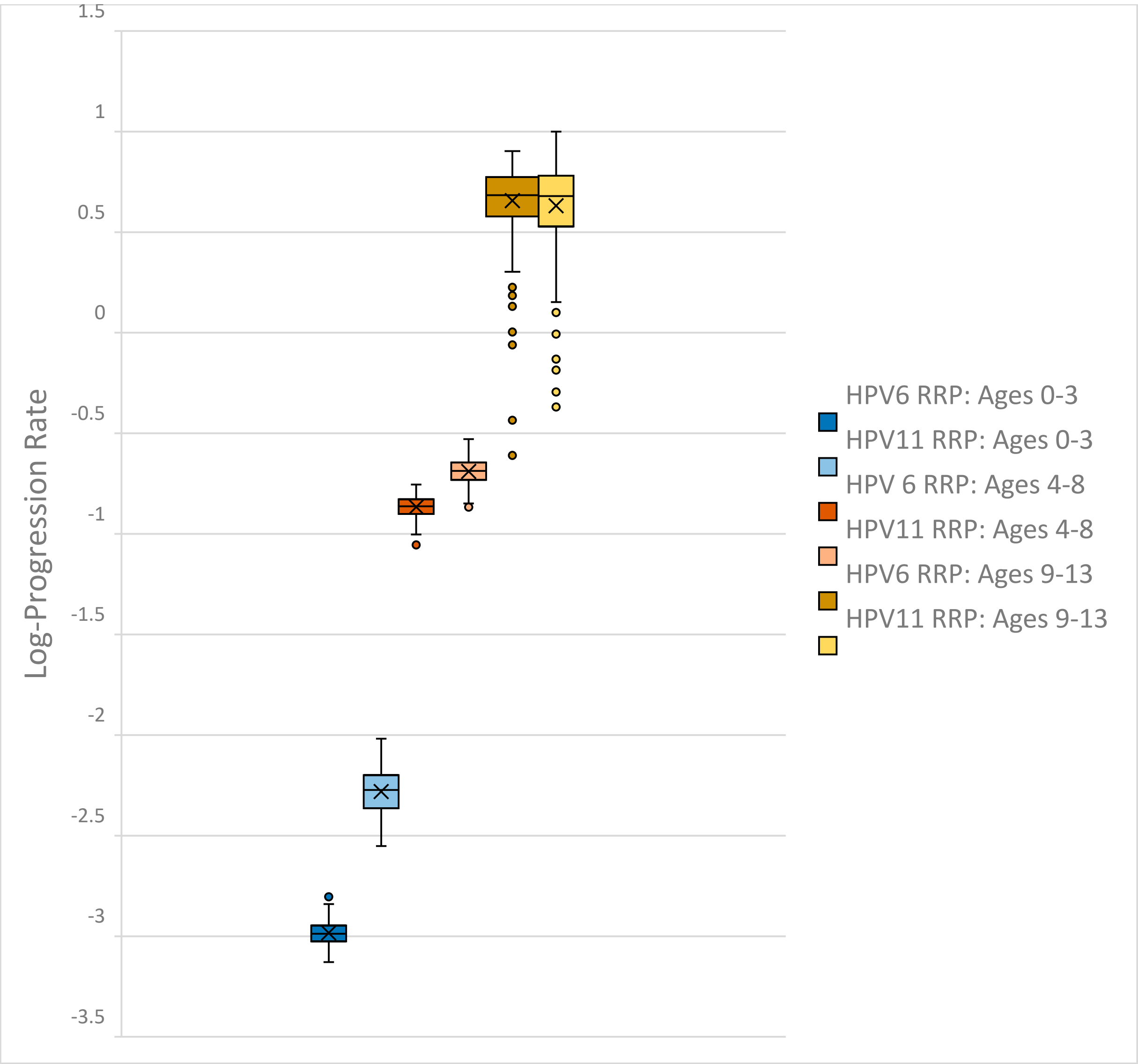

Figure 14: The log (base 10) of the calibrated progression rate to JORRP for the various Juvenile age groups.

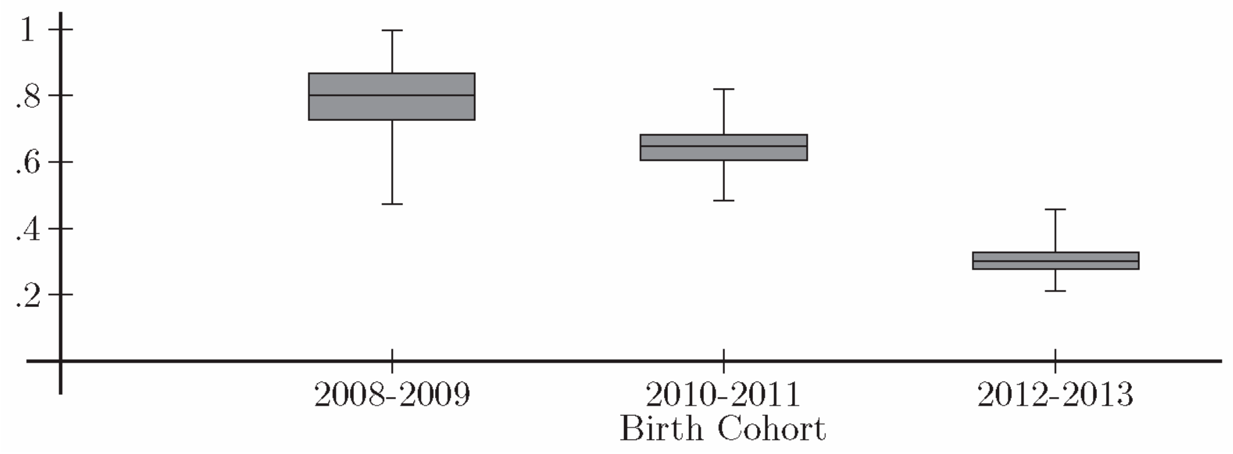

Figure 15: Proportion of the drop in observed birth-cohort JORRP incidence that can be attributed to vaccination of individuals over the age of 16 years. The last three birth cohorts are shown, as they are the cohorts that showed a significant drop in JORRP incidence.

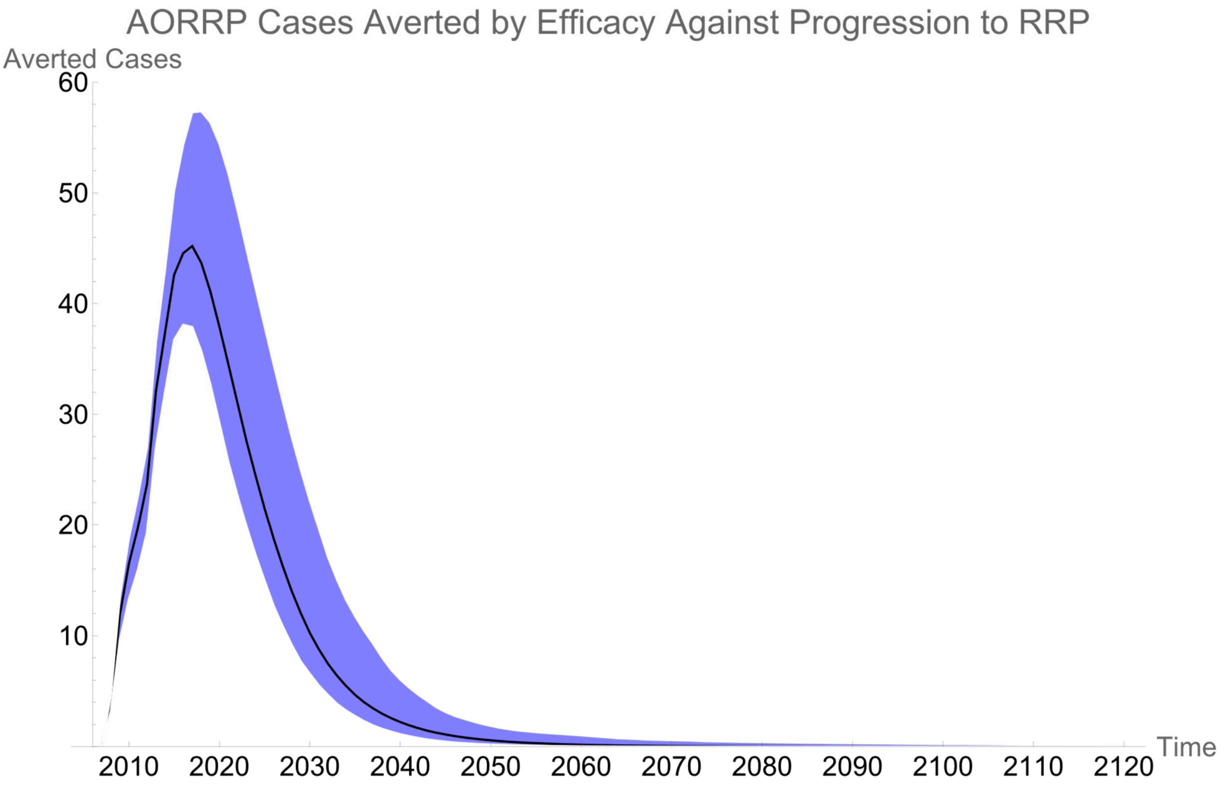

Figure 16:Cases of AORRP averted by assuming protection against progression to RRP.

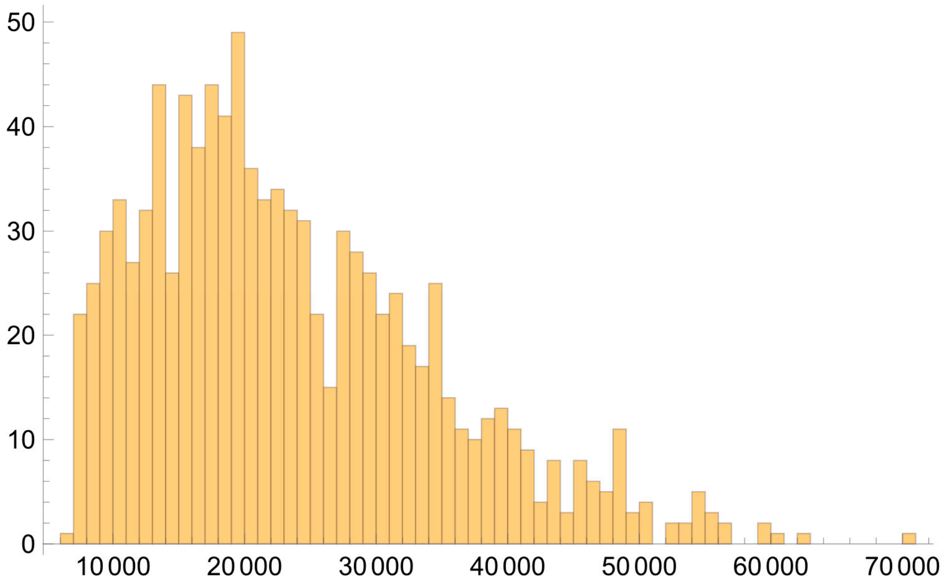

Figure 17: Sensitivity of QALYs saved from 2007 to 2021 to the utility associated with JORRP and AORRP. Utilities were drawn from Beta(5.7489,1.4761)

4 Bibliography

1. Cody P, Tobe K, Abe M, Elbasha EH. Public health impact and cost effectiveness of routine and catch-up vaccination of girls and women with a nine-valent HPV vaccine in Japan: a model-based study. BMC Infectious Diseases. 2021;21(1):11. doi: 10.1186/s12879-020-05632-0.
2. Daniels V, Prabhu VS, Palmer C, Samant S, Kothari S, Roberts C, et al. Public health impact and cost-effectiveness of catch-up 9-valent HPV vaccination of individuals through age 45 years in the United States. Human Vaccines & Immunotherapeutics. 2021:1-9. doi: 10.1080/21645515.2020.1852870.
3. Daniels V, Saxena K, Roberts C, Kothari S, Corman S, Yao L, et al. Impact of reduced human papillomavirus vaccination coverage rates due to COVID-19 in the United States: A model based analysis. Vaccine. 2021;39(20):2731-5.
4. Dasbach EJ, Insinga RP, Elbasha EH. The epidemiological and economic impact of a quadrivalent human papillomavirus vaccine (6/11/16/18) in the UK. BJOG : an international journal of obstetrics and gynaecology. 2008;115(8):947-56. doi: 10.1111/j.1471-0528.2008.01743.x. PubMed PMID: 18503574.
5. Elbasha EH, Dasbach EJ. Impact of vaccinating boys and men against HPV in the United States. Vaccine. 2010;28(42):6858-67. doi: 10.1016/j.vaccine.2010.08.030. PubMed PMID: 20713101.
6. Elbasha EH, Dasbach EJ, Insinga RP. Model for assessing human papillomavirus vaccination strategies. Emerging infectious diseases. 2007;13(1):28-41. doi: 10.3201/eid1301.060438. PubMed PMID: 17370513; PubMed Central PMCID: PMC2725801.
7. Hethcote HW. An age-structured model for pertussis transmission. Mathematical biosciences. 1997;145(2):89-136.
8. Meites E, Stone L, Amiling R, Singh V, Unger ER, Derkay C, et al. Significant Declines in Juvenile Onset Recurrent Respiratory Papillomatosis following HPV Vaccine Introduction in the United States. Clinical Infectious Diseases: an Official Publication of the Infectious Diseases Society of America. 2021.

9. Lewis RM, Markowitz LE. Human papillomavirus vaccination coverage among females and males, National Health and Nutrition Examination Survey, United States, 2007–2016. Vaccine. 2018;36(19):2567-73. doi: https://doi.org/10.1016/j.vaccine.2018.03.083.
10. Kung H-C, Hoyert DL, Xu J, Murphy SL. Deaths: final data for 2005. 2008.
11. Bureau USC. 2010 - 2018 National and State Population Estimates 2019 [cited 2021 8/10]. Available from: https://www.census.gov/newsroom/press-kits/2018/pop-estimates-national-state.html.
12. Mosher WD, Chandra A, Jones J. Sexual behavior and selected health measures: men and women 15-44 years of age, United States, 2002: US Department of Health and Human Services, Centers for Disease Control and ...; 2005.
13. Laumann EO, Gagnon JH, Michael RT, Michaels S. The social organization of sexuality: Sexual practices in the United States: University of Chicago press; 2000.
14. Abma JC. Sexual activity and contraceptive practices among teenagers in the United States, 1988 and 1995: United States Government Printing; 2001.
15. Martinez GM, Daniels K, Febo-Vazquez I. Fertility of Men and Women Aged 15-44 in the United States: National Survey of Family Growth, 2011-2015. National health statistics reports. 2018;(113):1-17.
16. Omland T, Akre H, Lie KA, Jebsen P, Sandvik L, Brøndbo K. Risk factors for aggressive recurrent respiratory papillomatosis in adults and juveniles. PLoS One. 2014;9(11):e113584.
17. Bishai D, Kashima H, Shah K. The Cost of Juvenile-Onset Recurrent Respiratory Papillomatosis. Archives of Otolaryngology–Head & Neck Surgery. 2000;126(8):935-9. doi: 10.1001/archotol.126.8.935.
18. Teen Vaccination Coverage Publications and Resources: Center for Disease Control; 2022 [cited 2022 April 18]. Available from: https://www.cdc.gov/vaccines/imz-managers/coverage/teenvaxview/pubs-presentations.html.
19. Bureau USC. National Intercensal Datasets 2000-2010 2022 [cited 2022 April 19]. Available from: https://www.census.gov/data/datasets/time-series/demo/popest/intercensal-2000-2010-national.html.
20. Amiling R, Meites E, Querec TD, Stone L, Singh V, Unger ER, et al. Juvenile-onset recurrent respiratory papillomatosis in the United States, epidemiology and HPV types—2015–2020. Journal of the Pediatric Infectious Diseases Society. 2021.
21. Gamerman D, Lopes HF. Markov chain Monte Carlo: stochastic simulation for Bayesian inference: CRC Press; 2006.
22. Dunne EF, Sternberg M, Markowitz LE, McQuillan G, Swan D, Patel S, et al. Human papillomavirus (HPV) 6, 11, 16, and 18 prevalence among females in the United States—National Health and Nutrition Examination Survey, 2003–2006: opportunity to measure HPV vaccine impact? Journal of Infectious Diseases. 2011;204(4):562-5.
23. Liu G, Markowitz LE, Hariri S, Panicker G, Unger ER. Seroprevalence of 9 human papillomavirus types in the United States, 2005–2006. The Journal of infectious diseases. 2016;213(2):191-8.
24. Derkay CS. Task force on recurrent respiratory papillomas: a preliminary report. Archives of Otolaryngology–Head & Neck Surgery. 1995;121(12):1386-91.
25. Boyle P, Parkin DM. Cancer registration: principles and methods. Statistical methods for registries. IARC Sci Publ. 1991;(95):126-58. Epub 1991/01/01. PubMed PMID: 1894318.
26. Syrjänen S, Rintala M, Sarkola M, Willberg J, Rautava J, Koskimaa H, et al. Oral Human Papillomavirus Infection in Children during the First 6 Years of Life, Finland. Emerging infectious diseases. 2021;27(3):759.
27. Ingles DJ, Lin H-Y, Fulp WJ, Sudenga SL, Lu B, Schabath MB, et al. An analysis of HPV infection incidence and clearance by genotype and age in men: The HPV Infection in Men (HIM) Study. Papillomavirus Research. 2015;1:126-35.

28. Goodman MT, Shvetsov YB, McDuffie K, Wilkens LR, Zhu X, Thompson PJ, et al. Prevalence, acquisition, and clearance of cervical human papillomavirus infection among women with normal cytology: Hawaii Human Papillomavirus Cohort Study. Cancer research. 2008;68(21):8813-24.
29. Giuliano AR, Viscidi R, Torres BN, Ingles DJ, Sudenga SL, Villa LL, et al. Seroconversion following anal and genital HPV infection in men: the HIM study. Papillomavirus research. 2015;1:109-15.
